# Differentiated associations of inflammatory indices with laboratory-defined organ injury/involvement and hospitalization length in pediatric respiratory tract infections

**DOI:** 10.3389/fped.2026.1804507

**Published:** 2026-07-16

**Authors:** Jing Wang, Ruotong Dong, Song Mao

**Affiliations:** Department of Pediatrics, Shanghai Sixth People’s Hospital Affiliated to Shanghai Jiao Tong University School of Medicine, Shanghai, China

**Keywords:** children, hospitalization length, inflammatory indices, laboratory-defined organ injury/involvement, respiratory tract infections

## Abstract

**Background:**

To analyze the associations of inflammatory indices with laboratory-defined organ injury/involvement and hospitalization length in pediatric respiratory tract infections (RTIs).

**Methods:**

In this study, logistic regression analyses were performed to examine the associations between inflammatory index levels and laboratory-defined cardiac injury, liver injury, and kidney involvement. Receiver operating characteristic (ROC) curve analyses were performed to evaluate the discriminative ability of inflammatory indices for laboratory-defined organ injury/involvement. Linear regression analyses were conducted to assess the associations between inflammatory index levels and hospitalization length.

**Results:**

Lower levels of neutrophils, C-reactive protein (CRP), Systemic immune inflammation index (SII), neutrophil-to-lymphocyte ratio (NLR), neutrophil-to-platelet ratio (NPR), and CRP-to-lymphocyte ratio (CLR) were associated with the presence of laboratory-defined cardiac injury. Higher lymphocyte levels and lower levels of neutrophils, platelets, CRP, SII, NLR, platelet-to-lymphocyte ratio (PLR), NPR, and CLR were associated with the presence of laboratory-defined liver injury. Higher levels of white blood cells (WBCs), neutrophils, CRP, SII, NLR, PLR, and CLR, together with lower lymphocyte levels, were associated with the presence of laboratory-defined kidney involvement. ROC curve analyses suggested that NLR, SII, and CLR had a relatively high discriminative ability for laboratory-defined cardiac injury; SII, NLR, and PLR for liver injury; and NLR, CLR, and NPR for laboratory-defined kidney involvement. Higher levels of WBCs, lymphocytes, CRP, NPR, and CLR, along with lower levels of platelets and Hb, were associated with longer hospitalization.

**Conclusion:**

Inflammatory indices, particularly composite indices, were associated with laboratory-defined organ injury/involvement and longer hospitalization in children with RTIs, suggesting their potential value as non-invasive exploratory markers. Future longitudinal studies are needed to confirm their prognostic utility.

## Introduction

Respiratory tract infections (RTIs) are among the most common diseases in children (1). Most pediatric RTIs have a favorable prognosis without serious complications or sequelae (2). However, organ injury such as cardiac, liver, and kidney injuries may occur during the course of RTIs. Although most organ injuries are self-limited, some may exert long-term adverse effects, especially in severe RTIs (3). For example, many adults with chronic kidney disease may have a history of kidney injury during childhood (4). Therefore, more attention should be paid to concurrent organ injury/involvement of pediatric RTIs. Identifying subgroups of children with RTIs who show a higher likelihood of organ injury/involvement or prolonged hospitalization may help improve clinical assessment and management.

Inflammation plays an important role in the development and progression of organ injury/involvement (5). Previous studies have also shown that inflammatory indices are associated with various comorbidities (6). Inflammation indicators can be categorized into two subgroups: single and composite indices, with the latter calculated based on multiple individual indicators. Systemic immune inflammation index (SII) and neutrophil-to-lymphocyte ratio (NLR) are common composite inflammatory indices (7). Derived from routine blood tests, these indices provide non-invasive and cost-effective measures for assessing systemic inflammation and immune status. Therefore, they may serve as simple exploratory indicators associated with organ injury/involvement and clinical course in children with RTIs.

In the past few years, several clinical prediction models have been developed to identify children at risk for RTI-related morbidity and mortality (8). However, most prediction models were based only on complex parameters, which may be time-consuming to obtain and not available in some primary healthcare institutions (8). Therefore, simple and feasible indicators for evaluating the prognosis of pediatric RTIs are of significant implications for timely and targeted clinical management. Routine blood tests are common and are easily performed, even in primary healthcare institutions.

The convenient sample taking, non-invasive nature, and short testing time make blood count–derived inflammatory indices potentially useful for clinical assessment. We, therefore, hypothesized that inflammatory indices derived from routine blood tests may be associated with laboratory-defined organ injury/involvement and hospitalization length in children with RTIs.

Based on these considerations, we performed this study to investigate the associations between blood count–derived inflammatory indices and laboratory-defined cardiac injury, liver injury, and kidney involvement, as well as hospitalization length, in children with RTIs. Specifically, we tested the following hypotheses: first, higher levels of inflammatory indices, particularly composite indices such as NLR, SII, and platelet-to-lymphocyte ratio (PLR), would be associated with increased odds of laboratory-defined cardiac injury, liver injury, and kidney involvement. Second, higher inflammatory indices would be associated with prolonged hospitalization. Third, composite inflammatory indices would show stronger associations with adverse clinical outcomes than single blood cell parameters, reflecting the combined effects of neutrophil-dominant inflammation, lymphocyte suppression, and platelet-related inflammatory activation. We also evaluated the discriminative ability of inflammatory indices for laboratory-defined organ injury/involvement using a receiver operating characteristic (ROC) curve analysis and examined potential threshold effects in the associations between inflammatory indices and hospitalization length.

## Methods

### Study design and population

We recruited hospitalized children with RTIs from the Pediatrics Department of Shanghai Sixth People's Hospital between January 2023 and June 2025. The pediatric RTIs were diagnosed as infections involving the sinuses, airway, throat, or lung (9). The children with RTIs were aged 0–18 years. All participants were generally healthy without chronic diseases, such as autoimmune and cardiopulmonary diseases, which might influence the recovery of RTIs. Meanwhile, patients with systemic diseases that targeted the heart, liver, and kidneys were excluded from the study.

Inclusion criteria required patients to have complete data on age, sex, preterm birth, hospitalization length, influenza A virus (FLUA), influenza B virus (FLUB), adenovirus, rhinovirus, RSV, MP, pneumonia, and cardiac, liver, and kidney injuries. Patients without these required data were excluded.

Our study was approved by the Ethics Committee of Shanghai Sixth People's Hospital (No. 2018-106). Our investigation was consistent with the Strengthening the Reporting of observational studies in Epidemiology (SROBE) reporting guidelines (10). The requirement of informed consent was waived because of the retrospective nature of the study. All the enrolled data were de-identified.

### Data collection

Respiratory pathogen infection (MP, FLUA, FLUB, adenovirus, rhinovirus, and RSV) was defined as a positive result from a multiplex real-time polymerase chain reaction assay using nasopharyngeal swab specimens. No viral culture was performed in this study. The clinical and laboratory data were retrospectively extracted from the electronical medical records of hospitalized inpatients. Meanwhile, the laboratory data were obtained from the first available test performed within 3 days after admission. We reviewed the data of age, sex, preterm birth, hospitalization length, pneumonia, serum creatine kinase MB form (CK-MB), alanine aminotransferase (ALT), aspartate aminotransferase (AST), C-reactive protein (CRP), interleukin 6 (IL-6), urine protein, MP, FLUA, FLUB, adenovirus, rhinovirus, and RSV.

Pneumonia diagnosis included non-pneumonia, ordinary pneumonia, and severe pneumonia (severe pneumonia is defined as pneumonia plus inability to drink, persistent vomiting, convulsions, lethargy, stridor at rest, severe malnutrition, or signs of respiratory failure, while ordinary pneumonia is defined as pneumonia without these plus symptoms) (11). Laboratory-defined cardiac injury was defined as serum CK-MB more than 25 U/L, laboratory-defined liver injury was defined as ALT or AST more than 40 U/L, and laboratory-defined kidney involvement was defined as a positive result of urine protein dipstick. These outcome definitions were based on routinely available laboratory indicators in the electronic medical records. Therefore, these outcomes should be interpreted as laboratory-defined organ injury or involvement rather than comprehensive clinically adjudicated organ dysfunction. Electrocardiography, echocardiography, and liver or kidney ultrasound examinations were not systematically available for all participants and were therefore not incorporated into the outcome definitions. Respiratory pathogen infection was defined as the positive result of nasopharyngeal swab.

### The definition and calculation of inflammatory indices

The inflammatory indices analyzed in this study included single inflammatory markers such as white blood cell (WBC) count (×10^9^/L), neutrophil count (×10^9^/L), lymphocyte count (×10^9^/L), platelet count (×10^9^/L), CRP level (mg/L), hemoglobin (Hb) level (g/L), and red blood cell distribution width (RDW) (%) and composite inflammatory indices were SII = platelet count×neutrophil count/lymphocyte count, NLR = neutrophil count/lymphocyte count, PLR = platelet count/lymphocyte count, NPR = neutrophil count/platelet count, and CLR = C-reactive protein level/lymphocyte count.

### Statistical analyses

Clinical and laboratory characteristics of the participants were reported according to laboratory-defined cardiac injury, liver injury, and kidney involvement status. Collinearity analyses of inflammatory indices and covariates were performed using variance inflation factors, with values <5 indicating no marked collinearity. Continuous variables were expressed as median (Q1, Q3), and categorical variables were expressed as numbers and percentages. Differences among groups were assessed using the rank-sum test for continuous variables and the *χ*^2^ test or Fisher's exact test for categorical variables.

We examined the association of inflammatory index levels with laboratory-defined cardiac injury, liver injury, and kidney involvement by using logistic regression analyses (Model I: no adjusted factors, Model II: adjusted for age, sex, and preterm birth, and Model III: adjusted for age, sex, preterm birth, FLUA, B, Adenovirus, Rhinovirus, RSV, MP, and pneumonia). Generalized additive models were used to visually assess the functional relationships between the levels of continuous inflammatory indices and laboratory-defined cardiac injury, liver injury, and kidney involvement. ROC curve analyses were performed to evaluate the discriminative ability of inflammatory indices for laboratory-defined cardiac injury, liver injury, and kidney involvement.

Linear regression analyses were used to assess the association between inflammatory index levels and hospitalization length (Model I: no adjusted factors, Model II: adjusted for age, sex, and preterm birth, and Model III: adjusted for age, sex, preterm birth, FLUA, B, Adenovirus, Rhinovirus, RSV, MP, and pneumonia). Threshold effects analyses were performed to investigate the associations between inflammatory index levels and hospitalization length. The infection point that provided the maximum model likelihood was identified. Restricted cubic spline was used to investigate the overall and non-linear relationship between inflammatory indices and hospitalization length.

Given that multiple inflammatory indices were evaluated across several clinical outcomes, the Benjamini–Hochberg false discovery rate (FDR) method was used to correct for multiple comparisons. Within each clinical outcome, FDR correction was applied to the *P* values for trend obtained from the fully adjusted Model III.

Associations that remained statistically significant after FDR correction were considered more robust, whereas associations significant only before correction were interpreted as exploratory.

The reference ranges of CK-MB, ALT, and AST may vary according to age in children, especially in younger children. Age-stratified sensitivity analyses were performed to assess the robustness of the main findings. The primary multivariable logistic regression analyses for laboratory-defined cardiac and liver injury were repeated within predefined pediatric age groups: <3 years, 3 to <6 years, 6 to <12 years, and ≥12 years. In addition, laboratory reference ranges may be more variable in younger children, and therefore, the analyses were repeated after excluding children younger than 3 years. These analyses were performed to evaluate whether the main associations were materially affected by age-related variation in laboratory reference ranges and should be interpreted as sensitivity analyses rather than definitive age-specific effect estimates.

The data analyses were performed using R software, version 4.2, provided by the R project for statistical computing, EmpowerStats (http://www.empowerstats.com, X&Y Solutions, Inc., Boston, MA), and the Free Statistics analysis platform (Version 2.0, Beijing, China, http://www.clinicalscientists.cn/freestatistics). A two-sided *p*-value less than 0.05 was considered statistically significant.

## Results

### Participants’ characteristics

A total of 1,046 pediatric RTI cases were included. The median age was 7 years, and 48.95% of the participants were female. Based on the predefined laboratory criteria, 188 children had laboratory-defined cardiac injury, 186 had laboratory-defined liver injury, and 185 had laboratory-defined kidney involvement. The baseline characteristics of the enrolled children are presented in Table 1. A collinearity analysis showed that the variance inflation factors of inflammatory indices and covariates were all <5 (Supplementary Table S1).

**Table 1:** Baseline characteristics of pediatric RTI based on laboratory-defined cardiac injury, liver injury, and kidney involvement.

Characteristics	All	Non-cardiac injury	Cardiac injury	Non-liver injury	Liver injury	Non-kidney involvement	Kidney involvement
	*n* = 1,046	*n* = 858	*n* = 188	*n* = 860	*n* = 186	*n* = 861	*n* = 185
Age (years)	7.0 (5.0, 10.0)	8.0 (6.0, 10.0)	5.0 (2.0, 7.0)	7.0 (6.0, 10.0)	6.0 (3.0, 9.0)	7.0 (5.0, 10.0)	8.0 (6.0, 10.0)
			*P* < 10^−3^		*P* < 10^−3^		*P* = 0.021
Age group							
age <3	77 (7.36%)	26 (3.03%)	51 (27.13%)	40 (4.65%)	37 (19.89%)	67 (7.78%)	10 (5.41%)
3 ≤ age <6	217 (20.75%)	155 (18.07%)	62 (32.98%)	167 (19.42%)	50 (26.88%)	191 (22.18%)	26 (14.05%)
6 ≤ age <12	627 (59.94%)	555 (64.69%)	72 (38.30%)	543 (63.14%)	84 (45.16%)	506 (58.77%)	121 (65.41%)
Age ≥12	125 (11.95%)	122 (14.22%)	3 (1.60%)	110 (12.79%)	15 (8.06%)	97 (11.27%)	28 (15.14%)
			*P* < 10^−3^		*P* < 10^−3^		*P* = 0.029
Gender							
Female	512 (48.95%)	431 (50.23%)	81 (43.09%)	430 (50.00%)	82 (44.09%)	406 (47.15%)	106 (57.30%)
Male	534 (51.05%)	427 (49.77%)	107 (56.91%)	430 (50.00%)	104 (55.91%)	455 (52.85%)	79 (42.70%)
			*P* = 0.076		*P* = 0.143		*P* = 0.012
Preterm birth							
No	930 (88.91%)	770 (89.74%)	160 (85.11%)	761 (88.49%)	169 (90.86%)	760 (88.27%)	170 (91.89%)
Yes	116 (11.09%)	88 (10.26%)	28 (14.89%)	99 (11.51%)	17 (9.14%)	101 (11.73%)	15 (8.11%)
			*P* = 0.067		*P* = 0.35		*P* = 0.155
FLUA							
No	1,014 (96.94%)	831 (96.85%)	183 (97.34%)	831 (96.63%)	183 (98.39%)	837 (97.21%)	177 (95.68%)
Yes	32 (3.06%)	27 (3.15%)	5 (2.66%)	29 (3.37%)	3 (1.61%)	24 (2.79%)	8 (4.32%)
			*P* = 0.725		*P* = 0.206		*P* = 0.271
FLUB							
No	1,022 (97.71%)	837 (97.55%)	185 (98.40%)	839 (97.56%)	183 (98.39%)	844 (98.03%)	178 (96.22%)
Yes	24 (2.29%)	21 (2.45%)	3 (1.60%)	21 (2.44%)	3 (1.61%)	17 (1.97%)	7 (3.78%)
			*P* = 0.480		*P* = 0.494		*P* = 0.136
Adenovirus							
No	957 (91.49%)	783 (91.26%)	174 (92.55%)	778 (90.47%)	179 (96.24%)	796 (92.45%)	161 (87.03%)
Yes	89 (8.51%)	75 (8.74%)	14 (7.45%)	82 (9.53%)	7 (3.76%)	65 (7.55%)	24 (12.97%)
			*P* = 0.565		*P* = 0.011		*P* = 0.016
Rhinovirus							
No	951 (90.92%)	784 (91.38%)	167 (88.83%)	779 (90.58%)	172 (92.47%)	777 (90.24%)	174 (94.05%)
Yes	95 (0.08%)	74 (8.62%)	21 (11.17%)	81 (9.42%)	14 (7.53%)	84 (9.76%)	11 (5.95%)
			*P* = 0.271		*P* = 0.416		*P* = 0.102
RSV							
No	988 (94.46%)	826 (96.27%)	162 (86.17%)	821 (95.47%)	167 (89.78%)	807 (93.73%)	181 (97.84%)
Yes	58 (5.54%)	32 (3.73%)	26 (13.83%)	39 (4.53%)	19 (10.22%)	54 (6.27%)	4 (2.16%)
			*P* < 0.001		*P* = 0.002		*P* = 0.027
MP							
No	534 (51.05%)	414 (48.25%)	120 (63.83%)	422 (49.07%)	112 (60.22%)	444 (51.57%)	90 (48.65%)
Yes	512 (48.95%)	444 (51.75%)	68 (36.17%)	438 (50.93%)	74 (39.78%)	417 (48.43%)	95 (51.35%)
			*P* < 0.001		*P* = 0.006		*P* = 0.471
Pneumonia							
No	227 (21.70%)	191 (22.26%)	36 (19.15%)	160 (18.60%)	67 (36.02%)	188 (21.84%)	39 (21.08%)
Ordinary	669 (63.96%)	542 (63.17%)	127 (67.55%)	581 (67.56%)	88 (47.31%)	567 (65.85%)	102 (55.14%)
Severe	150 (14.34%)	125 (14.57%)	25 (13.30%)	119 (13.84%)	31 (16.67%)	106 (12.31%)	44 (23.78%)
			*P* = 0.516		*P* < 0.001		*P* < 0.001

RTIs, respiratory tract infections; MP, *Mycoplasma pneumoniae*; RSV, respiratory syncytial virus; categorical variables: number (percentage), Chi-square test or Fisher's precision probability test (theoretical number <10).

Children with laboratory-defined cardiac injury were younger and had more RSV infections and fewer MP infections than those without laboratory-defined cardiac injury. Children with laboratory-defined liver injury were younger and had fewer adenovirus infections, MP infections, and pneumonia cases but more RSV infections than those without laboratory-defined liver injury. Children with laboratory-defined kidney involvement were older, constituted a higher proportion of females, and had adenovirus infection and severe pneumonia but fewer RSV infections than those without laboratory-defined kidney involvement (Table 1).

Children with laboratory-defined cardiac injury had lower levels of neutrophils, CRP, Hb, SII, NLR, PLR, NPR, and CLR, and higher levels of lymphocytes and RDW than those without laboratory-defined cardiac injury. Children with laboratory-defined liver injury had lower levels of neutrophils, platelets, CRP, SII, NLR, PLR, NPR, and CLR and higher levels of lymphocytes and RDW than those without laboratory-defined liver injury. Children with laboratory-defined kidney involvement had lower lymphocyte levels and higher levels of neutrophils, CRP, SII, NLR, PLR, NPR, and CLR than those without laboratory-defined kidney involvement (Table 2).

**Table 2:** Baseline differences in single and composite inflammatory indices between groups with and without laboratory-defined cardiac injury, liver injury, and kidney involvement.

Variables	Non-cardiac injury	Cardiac injury	Non-liver injury	Liver injury	Non-kidney involvement	Kidney involvement
	*n* = 858	*n* = 188	*n* = 860	*n* = 186	*n* = 861	*n* = 185
Single inflammatory indicators						
WBC (×10^9^/L)	7.3 (5.8–9.9)	7.4 (5.4–10.1)	7.3 (5.7–9.8)	7.6 (5.32–10.57)	7.3 (5.6–9.8)	8 (5.9–10.8)
		*P* = 0.626		*P* = 0.849		*P* = 0.077
Neutrophil (×10^9^/L)	4.2 (2.8–6.07)	3.1 (1.9–5.3)	4.2 (2.9–6.1)	2.85 (1.7–4.7)	4 (2.6–5.6)	4.8 (3.4–6.8)
		*P* < 0.001		*P* < 0.001		*P* < 0.001
Lymphocyte (×10^9^/L)	2.10 (1.60–2.90)	2.7 (2.00–4.03)	2.1 (1.6–2.9)	2.8 (1.83–5.8)	2.3 (1.7–3.1)	1.9 (1.4–2.5)
		*P* < 0.001		*P* < 0.001		*P* < 0.001
Platelet (×10^9^/L)	250 (199–313.75)	248 (192–310.25)	256 (205–321.5)	220.5 (169.25–283)	252 (200–312)	245 (189–314)
		*P* = 0.785		*P* < 0.001		*P* = 0.231
CRP (mg/L)	11.55 (6.16–25.44)	8.09 (1.77–13.68)	11.23 (6.12–24.68)	7.58 (2.43–15.03)	9.79 (4.18–19.97)	17.17 (7.11–36.96)
		*P* < 0.001		*P* < 0.001		*P* < 0.001
Hb (g/L)	126 (120–133)	125 (118–130)	126 (120–133)	126.5 (119.25–131)	126 (120–132)	126.66 (120–133)
		*P* = 0.004		*P* = 0.687		*P* = 0.865
RDW %	12.3 (11.90–12.80)	12.5 (12.1–13.03)	12.3 (11.9–12.8)	12.5 (12.1–13.07)	12.3 (11.9–12.8)	12.4 (11.9–12.9)
		*P* < 0.001		*P* = 0.003		*P* = 0.353
Composite inflammatory indices						
SII	512.98 (288.25–810.68)	273.53 (134.20–537.07)	515.45 (296.60–813.39)	197.89 (60.74–507.64)	439.70 (247.53–730.78)	590.15 (330.51–1,087.48)
		*P* < 0.001		*P* < 0.001		*P* < 0.001
NLR	1.98 (1.23–3.05)	1.11 (0.52–2.03)	1.98 (1.23–3.07)	0.9 (0.33–1.98)	1.76 (1.02–2.79)	2.5 (1.46–4.07)
		*P* < 0.001		*P* < 0.001		*P* < 0.001
PLR	118.42 (83.03–159.89)	87.88 (62.08–129.24)	118.55 (85.5–159.53)	79.01 (37.05–124.41)	108.39 (77.86–150)	125.33 (83.91–180)
		*P* < 0.001		*P* < 0.001		*P* = 0.003
NPR	0.02 (0.01–0.02)	0.01 (0.01–0.02)	0.02 (0.01–0.02)	0.01 (0.01–0.02)	0.02 (0.01–0.02)	0.02 (0.01–0.03)
		*P* < 0.001		*P* < 0.001		*P* < 0.001
CLR	5.72 (1.96–14.28)	2.54 (0.47–6.96)	10.20 (13.34) 5.63 (2.08–13.30)	2.30 (0.38–7.89)	4.33 (1.23–10.58)	9.58 (2.65–20.05)
		*P* < 0.001		*P* < 0.001		*P* < 0.001

WBC, white blood cell; CRP, C-reactive protein; HB, hemoglobin; RDW, red blood cell distribution width; SII, systemic immune-inflammation index; NLR, neutrophil-to-lymphocyte ratio; PLR, platelet-to-lymphocyte ratio; NPR, neutrophil-to-platelet ratio; CLR, CRP-to-lymphocyte ratio; median (Q1–Q3).

### Associations between inflammatory index quartiles and laboratory-defined cardiac injury, liver injury, and kidney involvement

For laboratory-defined cardiac injury, higher levels of neutrophils, CRP, SII, NLR, NPR, and CLR were associated with lower odds of laboratory-defined cardiac injury in the fully adjusted model (Supplementary Figure S1). Among these indices, the most representative associations were observed for NLR, SII, and CLR. Compared with the lowest quartile, the highest quartile of NLR was associated with lower odds of laboratory-defined cardiac injury (OR = 0.41, 95% CI: 0.25–0.69), as were SII (OR = 0.42, 95% CI: 0.25–0.71) and CLR (OR = 0.43, 95% CI: 0.25–0.75).

For laboratory-defined liver injury, higher levels of neutrophils, platelets, CRP, SII, NLR, PLR, NPR, and CLR were associated with lower odds of laboratory-defined liver injury in the fully adjusted model, whereas higher lymphocyte levels were associated with higher odds of laboratory-defined liver injury (Supplementary Figure S1). Representative associations included SII (Q4 vs. Q1: OR = 0.17, 95% CI: 0.10–0.29), NLR (Q4 vs. Q1: OR = 0.25, 95% CI: 0.15–0.41), PLR (Q4 vs. Q1: OR = 0.28, 95% CI: 0.17–0.46), and lymphocytes (Q4 vs. Q1: OR = 2.03, 95% CI: 1.23–3.35).

For laboratory-defined kidney involvement, higher levels of WBCs, neutrophils, CRP, SII, NLR, PLR, and CLR were associated with higher odds of laboratory-defined kidney involvement in the fully adjusted model, whereas higher lymphocyte levels were associated with lower odds of laboratory-defined kidney involvement (Supplementary Figure S1). Representative associations included NLR (Q4 vs. Q1: OR = 2.74, 95% CI: 1.67–4.48), CLR (Q4 vs. Q1: OR = 1.91, 95% CI: 1.18–3.09), NPR (Q4 vs. Q1: OR = 2.09, 95% CI: 0.83–5.23), and lymphocytes (Q4 vs. Q1: OR = 0.44, 95% CI: 0.26–0.73).

**Figure 1:**
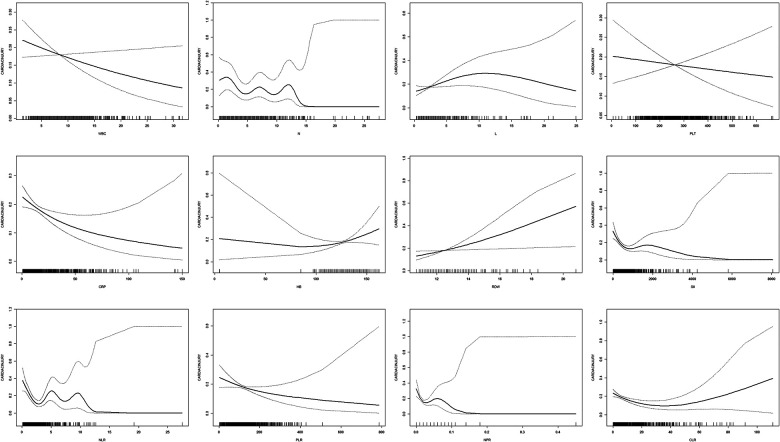
Relationship between the levels of continuous inflammatory indices and laboratory-defined cardiac injury.

**Figure 2:**
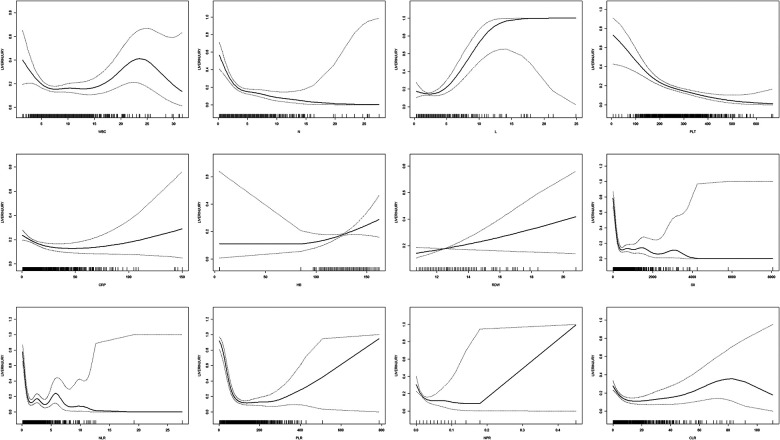
Relationship between the levels of continuous inflammatory indices and laboratory-defined liver injury.

**Figure 3:**
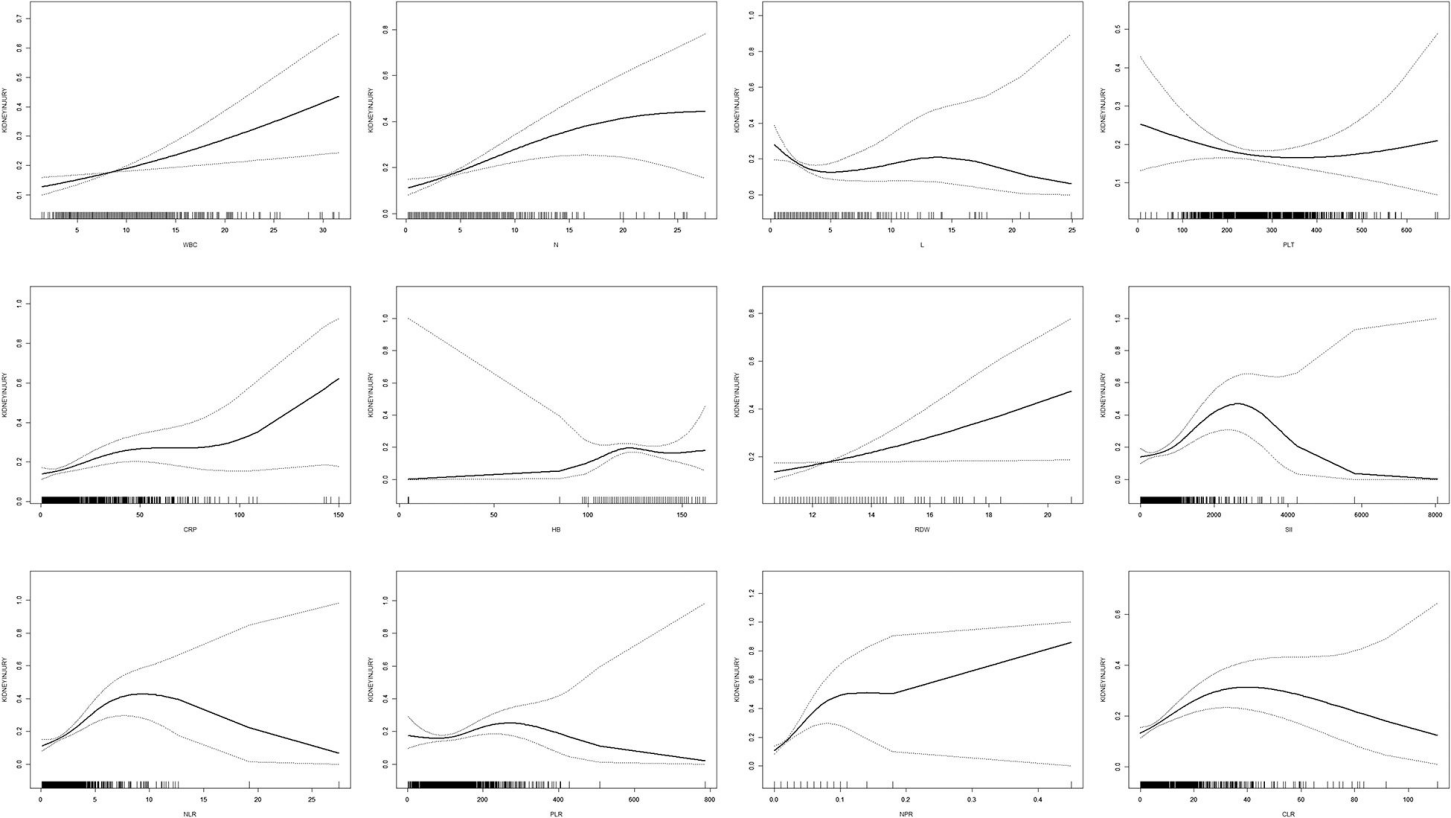
Relationship between the levels of continuous inflammatory indices and laboratory-defined kidney involvement.

The complete ORs and 95% CIs for all inflammatory indices across Models I–III are given in Table 3. Figures 1–3 show the relationships between continuous inflammatory index levels and laboratory-defined cardiac injury, liver injury, and kidney involvement.

**Table 3:** Association between inflammatory indices levels and laboratory-defined cardiac injury, liver injury, and kidney involvement.

Indicators	Q1	Q2	Q3	Q4	*P* for trend
WBC (N)	257	265	262	262	
Cardiac injury					
OR (95% CI) Model I	1	0.58 (0.36, 0.92)	0.84 (0.55, 1.30)	0.82 (0.53, 1.27)	0.714
OR (95% CI) Model II	1	0.65 (0.39, 1.07)	0.78 (0.49, 1.26)	0.63 (0.39, 1.01)	0.108
OR (95% CI) Model III	1	0.64 (0.39, 1.07)	0.81 (0.50, 1.31)	0.64 (0.39, 1.04)	0.150 *P*_FDR_: 0.164
Liver injury					
OR (95% CI) Model I	1	0.68 (0.43, 1.07)	0.77 (0.49, 1.21)	1.07 (0.70, 1.64)	0.615
OR (95% CI) Model II	1	0.71 (0.44, 1.14)	0.73 (0.46, 1.15)	0.96 (0.62, 1.48)	0.887
OR (95% CI) Model III	1	0.73 (0.45, 1.19)	0.74 (0.46, 1.21)	0.82 (0.51, 1.31)	0.451 *P*_FDR_: 0.451
Kidney involvement					
OR (95% CI) Model I	1	0.99 (0.62, 1.59)	1.19 (0.75, 1.88)	1.51 (0.96, 2.36)	0.047
OR (95% CI) Model II	1	0.95 (0.59, 1.53)	1.23 (0.77, 1.95)	1.64 (1.04, 2.59)	0.016
OR (95% CI) Model III	1	0.98 (0.60, 1.62)	1.24 (0.76, 2.01)	1.86 (1.15, 3.00)	0.006 *P*_FDR_: 0.009
Neutrophil (N)	257	266	257	266	
Cardiac injury					
OR (95% CI) Model I	1	0.46 (0.30, 0.70)	0.31 (0.20, 0.50)	0.41 (0.27, 0.63)	<0.001
OR (95% CI) Model II	1	0.59 (0.37, 0.94)	0.39 (0.24, 0.65)	0.48 (0.30, 0.77)	<0.001
OR (95% CI) Model III	1	0.58 (0.36, 0.93)	0.39 (0.23, 0.65)	0.47 (0.29, 0.76)	<0.001 *P*_FDR_: 0.003
Liver injury					
OR (95% CI) Model I	1	0.36 (0.23, 0.55)	0.30 (0.19, 0.47)	0.24 (0.15, 0.38)	<0.001
OR (95% CI) Model II	1	0.40 (0.26, 0.62)	0.33 (0.21, 0.52)	0.26 (0.16, 0.41)	<0.001
OR (95% CI) Model III	1	0.40 (0.25, 0.64)	0.33 (0.20, 0.54)	0.23 (0.14, 0.39)	<0.001 *P*_FDR_: 0.002
Kidney involvement					
OR (95% CI) Model I	1	1.09 (0.66, 1.80)	1.58 (0.99, 2.55)	2.04 (1.29, 3.22)	<0.001
OR (95% CI) Model II	1	1.01 (0.61, 1.67)	1.51 (0.94, 2.45)	2.02 (1.27, 3.21)	<0.001
OR (95% CI) Model III	1	0.99 (0.59, 1.66)	1.40 (0.85, 2.30)	2.00 (1.24, 3.22)	0.001 *P*_FDR_: 0.002
Lymphocyte (N)	252	247	281	266	
Cardiac injury					
OR (95% CI) Model I	1	0.98 (0.57, 1.71)	1.75 (1.07, 2.85)	3.25 (2.03, 5.19)	<0.001
OR (95% CI) Model II	1	0.88 (0.49, 1.56)	1.18 (0.70, 1.99)	1.44 (0.86, 2.43)	0.082
OR (95% CI) Model III	1	0.87 (0.49, 1.55)	1.19 (0.70, 2.03)	1.49 (0.87, 2.53)	0.066 *P*_FDR_: 0.088
Liver injury					
OR (95% CI) Model I	1	0.88 (0.52, 1.50)	1.17 (0.71, 1.91)	2.96 (1.89, 4.63)	<0.001
OR (95% CI) Model II	1	0.84 (0.49, 1.44)	1.01 (0.61, 1.67)	2.17 (1.35, 3.49)	<0.001
OR (95% CI) Model III	1	0.85 (0.49, 1.48)	0.99 (0.59, 1.67)	2.03 (1.23, 3.35)	0.002 *P*_FDR_: 0.003
Kidney involvement					
OR (95% CI) Model I	1	0.66 (0.43, 1.01)	0.51 (0.33, 0.78)	0.34 (0.21, 0.56)	<0.001
OR (95% CI) Model II	1	0.68 (0.45, 1.05)	0.52 (0.33, 0.80)	0.38 (0.23, 0.62)	<0.001
OR (95% CI) Model III	1	0.70 (0.46, 1.09)	0.54 (0.35, 0.85)	0.44 (0.26, 0.73)	<0.001 *P*_FDR_: 0.002
Platelet (N)	259	256	268	263	
Cardiac injury					
OR (95% CI) Model I	1	0.78 (0.50, 1.22)	0.87 (0.56, 1.34)	0.84 (0.54, 1.31)	0.557
OR (95% CI) Model II	1	0.76 (0.47, 1.24)	0.86 (0.54, 1.39)	0.74 (0.46, 1.20)	0.308
OR (95% CI) Model III	1	0.80 (0.49, 1.31)	0.89 (0.55, 1.45)	0.78 (0.47, 1.28)	0.411 *P*_FDR_: 0.411
Liver injury					
OR (95% CI) Model I	1	0.49 (0.32, 0.75)	0.53 (0.35, 0.80)	0.24 (0.14, 0.40)	<0.001
OR (95% CI) Model II	1	0.48 (0.31, 0.75)	0.50 (0.33, 0.77)	0.20 (0.12, 0.34)	<0.001
OR (95% CI) Model III	1	0.50 (0.32, 0.79)	0.51 (0.33, 0.81)	0.19 (0.11, 0.34)	<0.001 *P*_FDR_: 0.002
Kidney involvement					
OR (95% CI) Model I	1	0.75 (0.48, 1.17)	0.63 (0.40, 0.99)	0.83 (0.54, 1.28)	0.289
OR (95% CI) Model II	1	0.77 (0.49, 1.20)	0.64 (0.41, 1.01)	0.84 (0.54, 1.30)	0.321
OR (95% CI) Model III	1	0.75 (0.48, 1.19)	0.68 (0.42, 1.08)	0.85 (0.54, 1.34)	0.401 *P*_FDR_: 0.437
CRP (N)	261	262	261	262	
Cardiac injury					
OR (95% CI) Model I	1	0.72 (0.48, 1.09)	0.44 (0.28, 0.68)	0.31 (0.19, 0.51)	<0.001
OR (95% CI) Model II	1	0.82 (0.53, 1.28)	0.63 (0.39, 1.02)	0.49 (0.29, 0.82)	0.004
OR (95% CI) Model III	1	0.84 (0.54, 1.32)	0.64 (0.39, 1.05)	0.48 (0.28, 0.83)	0.005 *P*_FDR_: 0.010
Liver injury					
OR (95% CI) Model I	1	0.77 (0.51, 1.15)	0.37 (0.23, 0.59)	0.49 (0.31, 0.76)	<0.001
OR (95% CI) Model II	1	0.78 (0.51, 1.19)	0.42 (0.26, 0.68)	0.60 (0.38, 0.95)	0.003
OR (95% CI) Model III	1	0.70 (0.45, 1.10)	0.47 (0.28, 0.78)	0.54 (0.33, 0.89)	0.004 *P*_FDR_: 0.005
Kidney involvement					
OR (95% CI) Model I	1	0.96 (0.58, 1.61)	1.47 (0.91, 2.37)	2.48 (1.58, 3.90)	<0.001
OR (95% CI) Model II	1	0.93 (0.55, 1.56)	1.37 (0.84, 2.22)	2.26 (1.43, 3.59)	<0.001
OR (95% CI) Model III	1	0.86 (0.51, 1.46)	1.25 (0.76, 2.06)	1.96 (1.20, 3.19)	0.001 *P*_FDR_: 0.002
Hb (N)	251	246	260	289	
Cardiac injury					
OR (95% CI) Model I	1	1.00 (0.65, 1.54)	0.76 (0.49, 1.19)	0.58 (0.37, 0.92)	0.010
OR (95% CI) Model II	1	1.34 (0.83, 2.16)	1.24 (0.76, 2.03)	1.90 (1.12, 3.24)	0.036
OR (95% CI) Model III	1	1.31 (0.80, 2.14)	1.19 (0.72, 1.97)	1.81 (1.06, 3.12)	0.062 *P*_FDR_: 0.088
Liver injury					
OR (95% CI) Model I	1	0.82 (0.51, 1.31)	1.16 (0.75, 1.80)	0.80 (0.51, 1.25)	0.648
OR (95% CI) Model II	1	0.91 (0.57, 1.48)	1.51 (0.96, 2.38)	1.40 (0.86, 2.30)	0.056
OR (95% CI) Model III	1	1.06 (0.64, 1.76)	1.71 (1.06, 2.78)	1.61 (0.95, 2.73)	0.022 *P*_FDR_: 0.026
Kidney involvement					
OR (95% CI) Model I	1	1.21 (0.76, 1.92)	1.13 (0.71, 1.79)	1.07 (0.68, 1.68)	0.876
OR (95% CI) Model II	1	1.12 (0.70, 1.79)	1.02 (0.64, 1.63)	0.89 (0.55, 1.44)	0.556
OR (95% CI) Model III	1	1.23 (0.76, 1.99)	1.10 (0.68, 1.77)	0.98 (0.60, 1.59)	0.786 *P*_FDR_: 0.786
RDW (N)	197	266	293	290	
Cardiac injury					
OR (95% CI) Model I	1	0.89 (0.52, 1.54)	1.52 (0.92, 2.50)	2.04 (1.26, 3.32)	<0.001
OR (95% CI) Model II	1	0.89 (0.50, 1.60)	1.43 (0.84, 2.45)	1.52 (0.89, 2.59)	0.031
OR (95% CI) Model III	1	0.91 (0.50, 1.64)	1.45 (0.84, 2.50)	1.60 (0.94, 2.75)	0.020 *P*_FDR_: 0.034
Liver injury					
OR (95% CI) Model I	1	1.18 (0.70, 1.99)	1.26 (0.76, 2.10)	1.93 (1.18, 3.14)	0.005
OR (95% CI) Model II	1	1.20 (0.71, 2.05)	1.20 (0.72, 2.01)	1.63 (0.99, 2.70)	0.053
OR (95% CI) Model III	1	1.30 (0.75, 2.25)	1.10 (0.64, 1.88)	1.61 (0.96, 2.72)	0.113 *P*_FDR_: 0.123
Kidney involvement					
OR (95% CI) Model I	1	0.88 (0.53, 1.45)	1.17 (0.73, 1.89)	1.19 (0.74, 1.91)	0.246
OR (95% CI) Model II	1	0.87 (0.52, 1.44)	1.23 (0.76, 1.98)	1.28 (0.80, 2.08)	0.124
OR (95% CI) Model III	1	0.90 (0.54, 1.51)	1.29 (0.79, 2.10)	1.34 (0.82, 2.19)	0.100 *P*_FDR_: 0.120
SII (N)	262	261	261	262	
Cardiac injury					
OR (95% CI) Model I	1	0.42 (0.28, 0.64)	0.27 (0.17, 0.43)	0.26 (0.16, 0.41)	<0.001
OR (95% CI) Model II	1	0.59 (0.38, 0.93)	0.41 (0.25, 0.68)	0.44 (0.27, 0.73)	<0.001
OR (95% CI) Model III	1	0.58 (0.37, 0.93)	0.40 (0.24, 0.67)	0.42 (0.25, 0.71)	<0.001 *P*_FDR_: 0.003
Liver injury					
OR (95% CI) Model I	1	0.19 (0.12, 0.31)	0.15 (0.09, 0.25)	0.17 (0.11, 0.27)	<0.001
OR (95% CI) Model II	1	0.21 (0.13, 0.33)	0.17 (0.10, 0.28)	0.20 (0.12, 0.32)	<0.001
OR (95% CI) Model III	1	0.21 (0.13, 0.35)	0.17 (0.10, 0.29)	0.17 (0.10, 0.29)	<0.001 *P*_FDR_: 0.002
Kidney involvement					
OR (95% CI) Model I	1	1.11 (0.67, 1.83)	1.40 (0.86, 2.26)	2.40 (1.52, 3.77)	<0.001
OR (95% CI) Model II	1	1.01 (0.61, 1.67)	1.30 (0.80, 2.13)	2.20 (1.38, 3.49)	<0.001
OR (95% CI) Model III	1	1.00 (0.59, 1.68)	1.22 (0.73, 2.04)	2.10 (1.29, 3.41)	<0.001 *P*_FDR_: 0.002
NLR (N)	261	259	264	262	
Cardiac injury					
OR (95% CI) Model I	1	0.33 (0.22, 0.51)	0.22 (0.14, 0.35)	0.24 (0.15, 0.38)	<0.001
OR (95% CI) Model II	1	0.48 (0.30, 0.77)	0.37 (0.23, 0.62)	0.45 (0.27, 0.74)	<0.001
OR (95% CI) Model III	1	0.46 (0.29, 0.75)	0.35 (0.21, 0.59)	0.41 (0.25, 0.69)	<0.001 *P*_FDR_: 0.003
Liver injury					
OR (95% CI) Model I	1	0.24 (0.16, 0.38)	0.19 (0.12, 0.30)	0.20 (0.12, 0.32)	<0.001
OR (95% CI) Model II	1	0.27 (0.17, 0.42)	0.22 (0.14, 0.36)	0.24 (0.15, 0.39)	<0.001
OR (95% CI) Model III	1	0.31 (0.19, 0.50)	0.23 (0.14, 0.39)	0.25 (0.15, 0.41)	<0.001 *P*_FDR_: 0.002
Kidney involvement					
OR (95% CI) Model I	1	1.16 (0.69, 1.97)	1.54 (0.93, 2.54)	3.20 (2.02, 5.10)	<0.001
OR (95% CI) Model II	1	1.08 (0.64, 1.84)	1.41 (0.85, 2.35)	2.97 (1.84, 4.79)	<0.001
OR (95% CI) Model III	1	1.02 (0.59, 1.77)	1.24 (0.73, 2.11)	2.74 (1.67, 4.48)	<0.001 *P*_FDR_: 0.002
PLR (N)	262	260	262	262	
Cardiac injury					
OR (95% CI) Model I	1	0.72 (0.48, 1.08)	0.35 (0.22, 0.56)	0.33 (0.20, 0.53)	<0.001
OR (95% CI) Model II	1	0.92 (0.59, 1.43)	0.58 (0.35, 0.96)	0.70 (0.42, 1.18)	0.060
OR (95% CI) Model III	1	0.90 (0.58, 1.42)	0.58 (0.35, 0.97)	0.71 (0.41, 1.22)	0.075 *P*_FDR_: 0.090
Liver injury					
OR (95% CI) Model I	1	0.28 (0.18, 0.43)	0.21 (0.13, 0.33)	0.23 (0.15, 0.36)	<0.001
OR (95% CI) Model II	1	0.29 (0.19, 0.46)	0.24 (0.15, 0.38)	0.29 (0.18, 0.46)	<0.001
OR (95% CI) Model III	1	0.29 (0.18, 0.46)	0.23 (0.14, 0.38)	0.28 (0.17, 0.46)	<0.001 *P*_FDR_: 0.002
Kidney involvement					
OR (95% CI) Model I	1	1.14 (0.70, 1.86)	1.23 (0.76, 1.99)	2.11 (1.35, 3.31)	<0.001
OR (95% CI) Model II	1	1.10 (0.67, 1.80)	1.16 (0.71, 1.88)	1.90 (1.19, 3.02)	0.006
OR (95% CI) Model III	1	1.03 (0.62, 1.70)	1.06 (0.64, 1.75)	1.70 (1.05, 2.76)	0.028 *P*_FDR_: 0.037
NPR (N)	54	429	–	563	
Cardiac injury					
OR (95% CI) Model I	1	0.37 (0.21, 0.67)	–	0.20 (0.11, 0.36)	<0.001
OR (95% CI) Model II	1	0.58 (0.30, 1.11)	–	0.35 (0.18, 0.68)	<0.001
OR (95% CI) Model III	1	0.62 (0.32, 1.23)	–	0.36 (0.18, 0.72)	<0.001 *P*_FDR_: 0.003
Liver injury					
OR (95% CI) Model I	1	0.35 (0.20, 0.64)	–	0.24 (0.13, 0.44)	<0.001
OR (95% CI) Model II	1	0.44 (0.24, 0.80)	–	0.32 (0.17, 0.59)	0.002
OR (95% CI) Model III	1	0.38 (0.20, 0.74)	–	0.27 (0.14, 0.53)	0.001 *P*_FDR_: 0.002
Kidney involvement					
OR (95% CI) Model I	1	1.10 (0.45, 2.71)	–	2.33 (0.97, 5.57)	<0.001
OR (95% CI) Model II	1	0.98 (0.39, 2.42)	–	2.06 (0.85, 4.99)	<0.001
OR (95% CI) Model III	1	1.02 (0.40, 2.61)	–	2.09 (0.83, 5.23)	<0.001 *P*_FDR_: 0.002
CLR (N)	262	261	261	262	
Cardiac injury					
OR (95% CI) Model I	1	0.68 (0.45, 1.01)	0.39 (0.25, 0.61)	0.26 (0.16, 0.42)	<0.001
OR (95% CI) Model II	1	0.74 (0.48, 1.14)	0.59 (0.36, 0.96)	0.44 (0.26, 0.76)	0.002
OR (95% CI) Model III	1	0.74 (0.47, 1.15)	0.60 (0.37, 0.98)	0.43 (0.25, 0.75)	0.002 *P*_FDR_: 0.005
Liver injury					
OR (95% CI) Model I	1	0.52 (0.34, 0.78)	0.26 (0.16, 0.42)	0.36 (0.23, 0.57)	<0.001
OR (95% CI) Model II	1	0.52 (0.34, 0.79)	0.30 (0.18, 0.49)	0.45 (0.29, 0.72)	<0.001
OR (95% CI) Model III	1	0.53 (0.34, 0.82)	0.34 (0.20, 0.56)	0.43 (0.26, 0.71)	<0.001 *P*_FDR_: 0.002
Kidney involvement					
OR (95% CI) Model I	1	0.67 (0.39, 1.14)	1.49 (0.93, 2.37)	2.47 (1.59, 3.85)	<0.001
OR (95% CI) Model II	1	0.65 (0.38, 1.12)	1.41 (0.88, 2.26)	2.27 (1.44, 3.57)	<0.001
OR (95% CI) Model III	1	0.61 (0.35, 1.05)	1.28 (0.79, 2.08)	1.91 (1.18, 3.09)	<0.001 *P*_FDR_: 0.002

N, number; CRP, C-reactive protein; Hb, hemoglobin; RDW, red blood cell distribution width; AST, aspartate aminotransferase; SII, systemic immune-inflammation index; NLR, neutrophil-to-lymphocyte ratio; PLR, platelet-to-lymphocyte ratio; NPR, neutrophil-to-platelet ratio; CLR, CRP-to-lymphocyte ratio; OR, odds ratio; CI, confidence interval; Model I, no adjusted factors; Model II, adjusted for age, gender, and preterm birth; Model III, adjusted for age, gender, and preterm birth; FDR, false discovery rate. *P*-values reported as <0.001 were conservatively entered as 0.001 for FDR correction.

After applying the Benjamini–Hochberg FDR correction, the associations between inflammatory indices and laboratory-defined cardiac injury, liver injury, and kidney involvement that were significant before correction remained statistically significant. Therefore, the main findings were consistent after correction for multiple comparisons. The complete nominal and FDR-adjusted *P* values are presented in Table 3.

### Discriminative ability of inflammatory indices for laboratory-defined cardiac injury, liver injury, and kidney involvement

**Figure 4:**
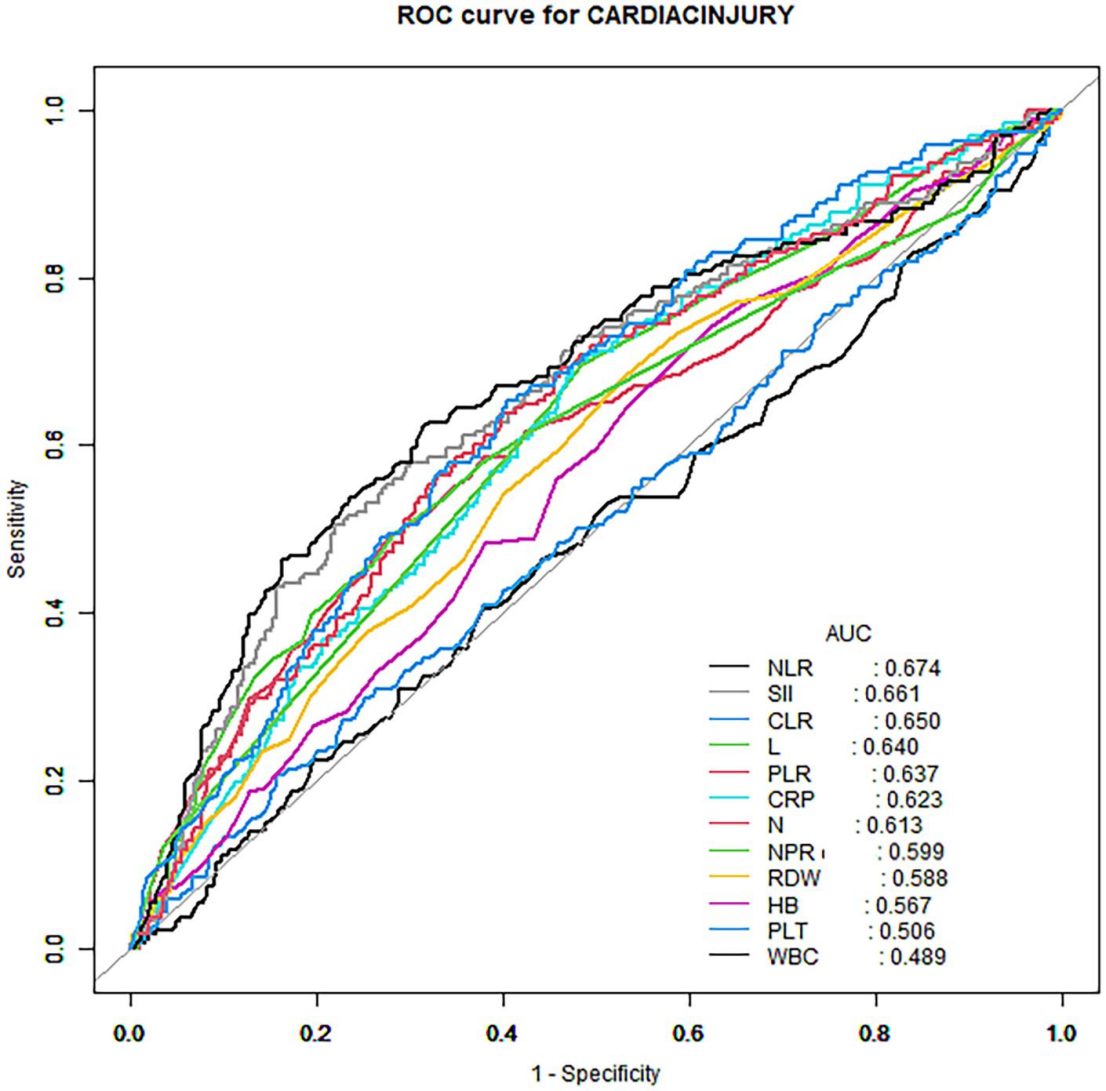
An ROC curve of inflammatory indices for laboratory-defined cardiac injury.

In ROC curve analyses, NLR, SII, and CLR showed the three highest AUCs for laboratory-defined cardiac injury, with AUCs of 0.67 (95% CI: 0.63–0.72), 0.66 (95% CI: 0.62–0.71), and 0.65 (95% CI: 0.61–0.69), respectively (Table 4, Figure 4).

**Table 4:** Discriminative ability of inflammatory indices for laboratory-defined cardiac injury, liver injury, and kidney involvement.

Inflammatory indicators	AUC	95% CI lower	95% CI upper	Sensitivity	Specificity	Accuracy
Cardiac injury						
WBC	0.49	0.44	0.54	0.22	0.80	0.70
Neutrophil	0.61	0.57	0.66	0.49	0.72	0.68
Lymphocyte	0.64	0.60	0.68	0.70	0.52	0.55
Platelet	0.51	0.46	0.55	0.21	0.84	0.73
CRP	0.62	0.58	0.67	0.70	0.52	0.55
Hb	0.57	0.52	0.61	0.74	0.38	0.44
RDW	0.59	0.54	0.63	0.68	0.47	0.51
SII	0.66	0.62	0.71	0.51	0.78	0.73
NLR	0.67	0.63	0.72	0.63	0.68	0.67
PLR	0.64	0.59	0.68	0.64	0.60	0.61
NPR	0.60	0.55	0.64	0.62	0.57	0.58
CLR	0.65	0.61	0.69	0.65	0.60	0.61
Liver injury						
WBC	0.50	0.45	0.55	0.39	0.69	0.64
Neutrophil	0.67	0.62	0.71	0.55	0.73	0.70
Lymphocyte	0.64	0.59	0.69	0.33	0.94	0.83
Platelet	0.63	0.59	0.68	0.53	0.67	0.64
CRP	0.60	0.55	0.65	0.50	0.70	0.66
Hb	0.49	0.45	0.54	0.47	0.56	0.54
RDW	0.57	0.52	0.61	0.52	0.59	0.58
SII	0.73	0.68	0.77	0.54	0.85	0.80
NLR	0.71	0.66	0.75	0.49	0.89	0.82
PLR	0.69	0.64	0.74	0.60	0.73	0.71
NPR	0.59	0.54	0.63	0.57	0.56	0.56
CLR	0.63	0.59	0.68	0.49	0.75	0.71
Kidney involvement						
WBC	0.54	0.49	0.59	0.55	0.55	0.55
Neutrophil	0.60	0.55	0.64	0.72	0.43	0.48
Lymphocyte	0.60	0.56	0.65	0.73	0.44	0.50
Platelet	0.53	0.48	0.58	0.34	0.73	0.66
CRP	0.60	0.55	0.65	0.53	0.67	0.65
Hb	0.50	0.46	0.55	0.91	0.13	0.27
RDW	0.52	0.47	0.57	0.61	0.45	0.48
SII	0.60	0.55	0.65	0.52	0.65	0.62
NLR	0.63	0.58	0.67	0.42	0.78	0.72
PLR	0.57	0.52	0.62	0.32	0.83	0.74
NPR	0.61	0.57	0.66	0.69	0.49	0.53
CLR	0.62	0.57	0.67	0.59	0.66	0.65

ROC, receiver operating curve; AUC, area under the curve; WBC, white blood cell; CRP, C-reactive protein; Hb, hemoglobin; RDW, red blood cell distribution width; SII, systemic immune-inflammation index; NLR, neutrophil-to-lymphocyte ratio; PLR, platelet-to-lymphocyte ratio; NPR, neutrophil-to-platelet ratio; CLR, CRP-to-lymphocyte ratio.

**Figure 5:**
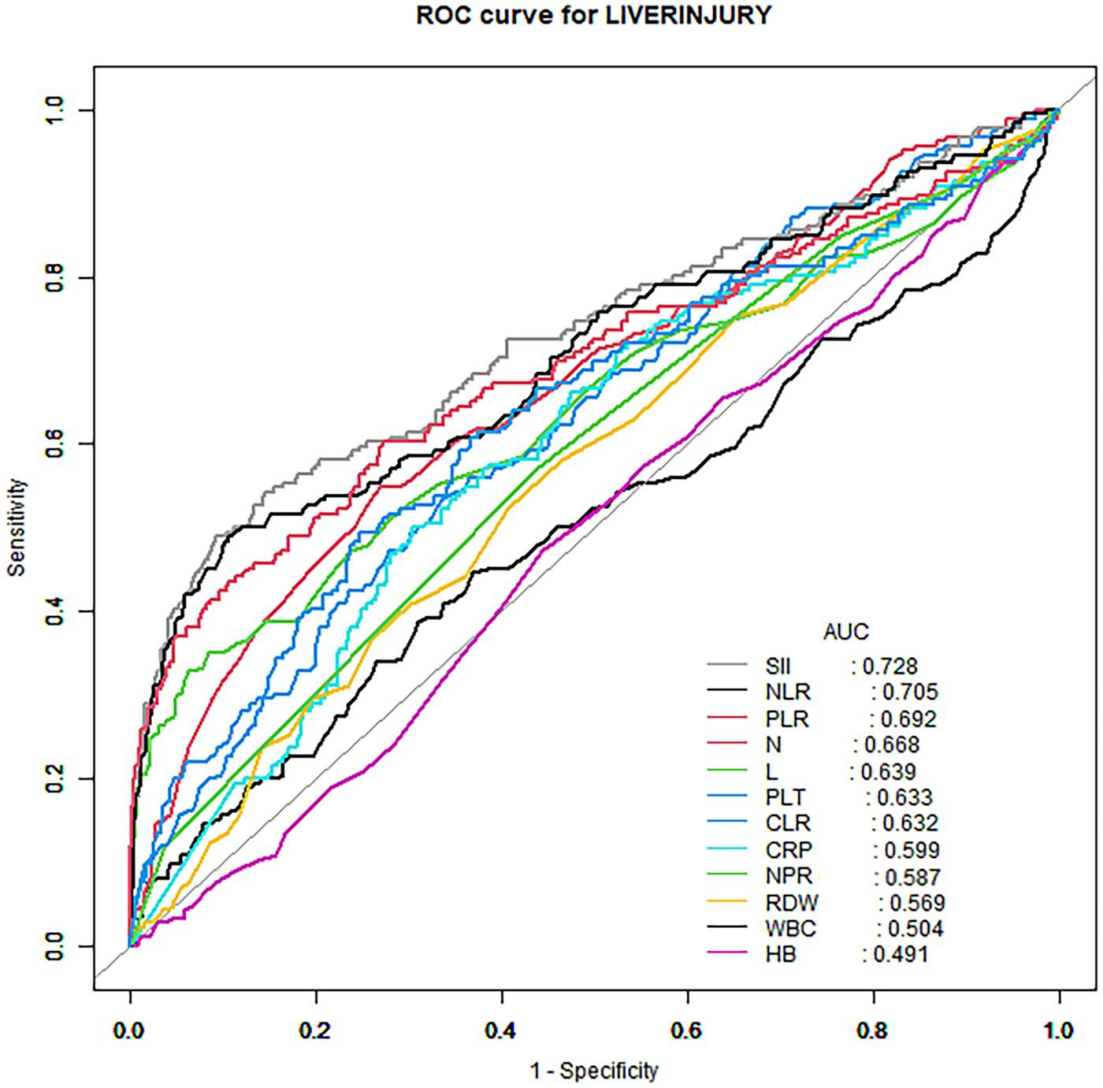
An ROC curve of inflammatory indices for laboratory-defined liver injury.

SII, NLR, and PLR showed the three highest AUCs for laboratory-defined liver injury, with AUCs of 0.73 (95% CI: 0.68–0.77), 0.71 (95% CI: 0.66–0.75), and 0.69 (95% CI: 0.64–0.74), respectively (Table 4, Figure 5).

**Figure 6:**
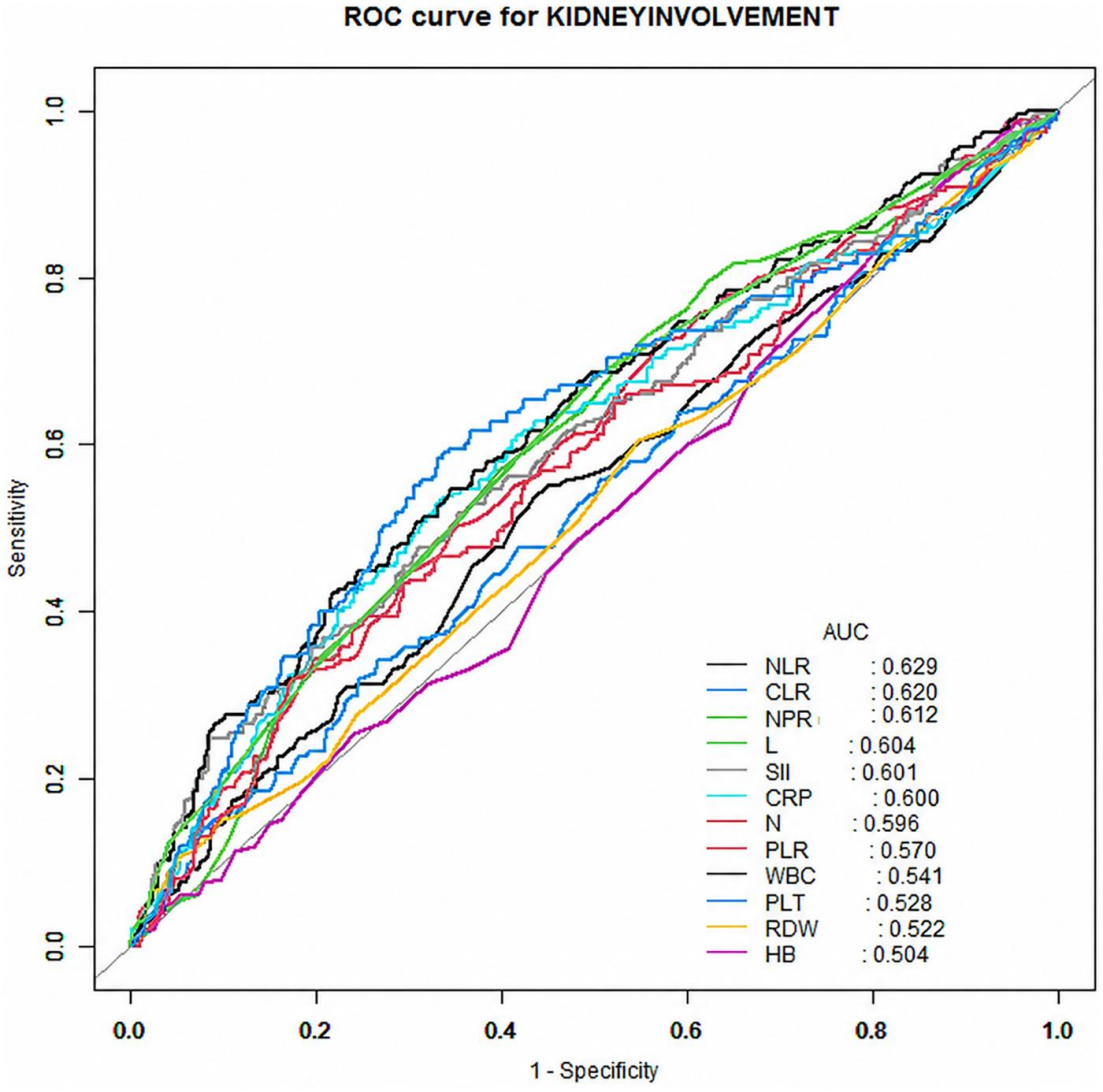
An ROC curve of inflammatory indices for laboratory-defined kidney involvement.

NLR, CLR, and NPR showed the three highest AUCs for laboratory-defined kidney involvement, with AUCs of 0.63 (95% CI: 0.58–0.67), 0.62 (95% CI: 0.57–0.67), and 0.61 (95% CI: 0.57–0.66), respectively (Table 4, Figure 6).

### Association between inflammatory index levels and hospitalization length

WBC level was positively associated with hospitalization length (*p* for trend: 0.011, continuous coef 95% CI: 0.03–0.09, Table 5). Lymphocyte level was positively associated with hospitalization length (*p* for trend: 0.673, continuous coef 95% CI: 0.10–0.21, Table 5). Platelet level was negatively associated with hospitalization length (*p* for trend: 0.036, continuous coef 95% CI: −0.00–−0.00, Table 5). CRP level was positively associated with hospitalization length (*p* for trend: <0.001, continuous coef 95% CI: 0.00–0.01, Table 5). Hb level was negatively associated with hospitalization length (*p* for trend: 0.005, continuous coef 95% CI: −0.02–−0.00, Table 5). NPR and CLR levels were positively associated with hospitalization length (*p* for trend: 0.002, continuous coef 95% CI: −3.99–8.52, Table 5; *p* for trend: 0.003, continuous coef 95% CI: −0.00–0.02, Table 5). Supplementary Figure S2 shows the overall and non-linear relationships between inflammatory index levels and hospitalization length.

**Table 5:** Association between inflammatory indices levels and hospitalization length.

Indicators	Q1	Q2	Q3	Q4	*P* for trend	Continuous
WBC (N)	257	265	262	262		
Coef (95% CI) Model I	0	0.58 (0.20, 0.95)	0.51 (0.14, 0.89)	0.50 (0.12, 0.87)	0.021	0.05 (0.02, 0.08)
Coef (95% CI) Model II	0	0.54 (0.17, 0.92)	0.51 (0.13, 0.89)	0.53 (0.15, 0.91)	0.011	0.06 (0.03, 0.09)
Coef (95% CI) Model III	0	0.47 (0.11, 0.83)	0.38 (0.02, 0.74)	0.54 (0.17, 0.90)	0.011 *P*_FDR_: 0.026	0.06 (0.03, 0.09)
Neutrophil (N)	257	266	257	266		
Coef (95% CI) Model I	0	0.37 (0.00, 0.75)	0.67 (0.29, 1.05)	0.38 (0.01, 0.76)	0.019	0.02 (−0.02, 0.06)
Coef (95% CI) Model II	0	0.34 (−0.04, 0.71)	0.63 (0.25, 1.01)	0.39 (0.01, 0.76)	0.019	0.03 (−0.01, 0.07)
Coef (95% CI) Model III	0	0.16 (−0.20, 0.53)	0.41 (0.04, 0.78)	0.26 (−0.10, 0.62)	0.088 *P*_FDR_: 0.151	0.02 (−0.02, 0.06)
Lymphocyte (N)	252	247	281	266		
Coef (95% CI) Model I	0	0.02 (−0.37, 0.41)	−0.42 (−0.80, −0.05)	−0.22 (−0.60, 0.16)	0.069	0.11 (0.06, 0.17)
Coef (95% CI) Model II	0	0.04 (−0.34, 0.43)	−0.39 (−0.77, −0.02)	−0.14 (−0.54, 0.25)	0.167	0.13 (0.07, 0.19)
Coef (95% CI) Model III	0	0.08 (−0.28, 0.45)	−0.24 (−0.60, −0.12)	0.03 (−0.35, 0.41)	0.673 *P*_FDR_: 0.734	0.15 (0.10, 0.21)
Platelet (N)	259	256	268	263		
Coef (95% CI) Model I	0	−0.14 (−0.52, 0.24)	−0.02 (−0.39, 0.36)	−0.17 (−0.55, 0.20)	0.522	−0.00 (−0.00, 0.00)
Coef (95% CI) Model II	0	−0.10 (−0.48, 0.28)	−0.03 (−0.40, 0.35)	−0.19 (−0.57, 0.19)	0.410	−0.00 (−0.00, 0.00)
Coef (95% CI) Model III	0	−0.07 (−0.43, 0.29)	−0.11 (−0.46, 0.25)	−0.40 (−0.76, −0.04)	0.036 *P*_FDR_: 0.072	−0.00 (−0.00, −0.00)
CRP (N)	261	262	261	262		
Coef (95% CI) Model I	0	0.25 (−0.12, 0.63)	0.64 (0.26, 1.01)	0.59 (0.22, 0.97)	<0.001	0.01 (0.00, 0.01)
Coef (95% CI) Model II	0	0.19 (−0.18, 0.57)	0.56 (0.19, 0.94)	0.51 (0.13, 0.90)	0.002	0.01 (−0.00, 0.01)
Coef (95% CI) Model III	0	0.08 (−0.28, 0.44)	0.58 (0.21, 0.94)	0.50 (0.13, 0.88)	<0.001 *P*_FDR_: 0.012	0.01 (0.00, 0.01)
Hb (N)	251	246	260	289		
Coef (95% CI) Model I	0	−0.12 (−0.50, 0.27)	−0.21 (−0.59, 0.17)	−0.40 (−0.77, −0.03)	0.030	−0.01 (−0.02, 0.00)
Coef (95% CI) Model II	0	−0.19 (−0.57, 0.20)	−0.30 (−0.68, 0.09)	−0.58 (−0.97, −0.19)	0.004	−0.01 (−0.02, −0.00)
Coef (95% CI) Model III	0	−0.08 (−0.45, 0.29)	−0.25 (−0.61, 0.12)	−0.52 (−0.89, −0.14)	0.005 *P*_FDR_: 0.015	−0.01 (−0.02, −0.00)
RDW (N)	197	266	293	290		
Coef (95% CI) Model I	0	−0.23 (−0.63, 0.18)	0.03 (−0.37, 0.43)	−0.29 (−0.69, 0.10)	0.343	−0.06 (−0.21, 0.08)
Coef (95% CI) Model II	0	−0.23 (−0.63, 0.18)	0.05 (−0.35, 0.44)	−0.28 (−0.68, 0.12)	0.398	−0.06 (−0.20, 0.09)
Coef (95% CI) Model III	0	−0.03 (−0.41, 0.35)	0.16 (−0.21, 0.54)	−0.05 (−0.43, 0.33)	0.958 *P*_FDR_: 0.958	−0.00 (−0.14, 0.14)
SII (N)	262	261	261	262		
Coef (95% CI) Model I	0	0.29 (−0.08, 0.67)	0.36 (−0.02, 0.74)	0.56 (0.19, 0.94)	0.004	0.00 (0.00, 0.00)
Coef (95% CI) Model II	0	0.22 (−0.16, 0.60)	0.31 (−0.07, 0.69)	0.49 (0.11, 0.88)	0.011	0.00 (−0.00, 0.00)
Coef (95% CI) Model III	0	0.08 (−0.29, 0.45)	0.08 (−0.29, 0.45)	0.20 (−0.17, 0.57)	0.317 *P*_FDR_: 0.423	0.00 (−0.00, 0.00)
NLR (N)	261	259	264	262		
Coef (95% CI) Model I	0	0.20 (−0.18, 0.57)	0.27 (−0.10, 0.65)	0.57 (0.19, 0.95)	0.003	0.06 (−0.01, 0.12)
Coef (95% CI) Model II	0	0.13 (−0.26, 0.51)	0.21 (−0.17, 0.60)	0.51 (0.12, 0.90)	0.009	0.05 (−0.01, 0.12)
Coef (95% CI) Model III	0	0.00 (−0.37, 0.37)	−0.03 (−0.40, 0.34)	0.32 (−0.06, 0.69)	0.111 *P*_FDR_: 0.167	0.04 (−0.02, 0.10)
PLR (N)	262	260	262	262		
Coef (95% CI) Model I	0	−0.35 (−0.73, 0.03)	−0.06 (−0.44, 0.31)	0.30 (−0.08, 0.67)	0.052	0.00 (−0.00, 0.00)
Coef (95% CI) Model II	0	−0.37 (−0.75, 0.00)	−0.12 (−0.50, 0.26)	0.20 (−0.18, 0.59)	0.171	0.00 (−0.00, 0.00)
Coef (95% CI) Model III	0	−0.47 (−0.82, −0.11)	−0.31 (−0.67, 0.06)	−0.15 (−0.53, 0.23)	0.632 *P*_FDR_: 0.734	−0.00 (−0.00, 0.00)
NPR (N)	54	429	–	563		
Coef (95% CI) Model I	0	0.81 (0.19, 1.43)	–	1.11 (0.50, 1.72)	<0.001	1.93 (−4.63, 8.50)
Coef (95% CI) Model II	0	0.74 (0.12, 1.36)	–	1.05 (0.43, 1.67)	0.001	2.32 (−4.23, 8.87)
Coef (95% CI) Model III	0	0.58 (−0.01, 1.18)	–	0.88 (0.29, 1.47)	0.002 *P*_FDR_: 0.012	2.27 (−3.99, 8.52)
CLR (N)	262	261	261	262		
Coef (95% CI) Model I	0	0.10 (−0.28, 0.47)	0.44 (0.07, 0.82)	0.57 (0.19, 0.94)	<0.001	0.01 (0.00, 0.02)
Coef (95% CI) Model II	0	0.06 (−0.31, 0.44)	0.38 (0.00, 0.76)	0.48 (0.10, 0.87)	0.005	0.01 (0.00, 0.02)
Coef (95% CI) Model III	0	−0.03 (−0.39, 0.32)	0.36 (0.00, 0.73)	0.46 (0.09, 0.84)	0.003 *P*_FDR_: 0.012	0.01 (−0.00, 0.02)

N, number; CRP, C-reactive protein; Hb, hemoglobin; RDW, red blood cell distribution width; AST, aspartate aminotransferase; SII, systemic immune-inflammation index; NLR, neutrophil-to-lymphocyte ratio; PLR, platelet-to-lymphocyte ratio; NPR, neutrophil-to-platelet ratio; CLR, CRP-to-lymphocyte ratio; Coef, coefficient; CI, confidence interval; Model I, no adjusted factors; Model II, adjusted for age, gender, and preterm birth, Model III, adjusted for age, gender, and preterm birth; FDR, false discovery rate. *P*-values reported as <0.001 were conservatively entered as 0.001 for FDR correction.

For hospitalization length, the associations of WBC/CRP/Hb//NPR/CLR remained significant after FDR correction, whereas some nominally significant association of PLT was attenuated after correction. The complete results are presented in Table 5.

### Threshold effects of inflammatory index levels on hospitalization length

Inflection points were observed in the associations of neutrophils, lymphocytes, CRP, SII, NLR, PLR, NPR, and CLR with hospitalization length (Supplementary Table S2). Significant associations were observed before and/or after these inflection points.

### Age-stratified sensitivity analyses

Overall, the main findings were generally consistent with the primary analyses, particularly after excluding children younger than 3 years. Among children aged 3 years or older, the representative associations of NLR, SII, and CLR with laboratory-defined cardiac injury, as well as those of SII, NLR, PLR, and lymphocytes with laboratory-defined liver injury, remained similar to the primary results. Some estimates in individual age strata were unstable, with wide confidence intervals or unavailable estimates due to sparse data; therefore, these age-stratified results were interpreted as sensitivity analyses rather than definitive age-specific findings. The detailed results are presented in Supplementary Table S3.

## Discussion

RTIs, which are major health concerns among children across the world, caused significant morbidity and even resulted in death in some severe cases (12). Precise and targeted intervention for different patient populations is vital for the improvement of overall prognosis of RTIs. Hence, identifying easily available inflammatory markers associated with laboratory-defined organ injury/involvement and longer hospitalization may provide useful information for clinical assessment. We explored the associations between inflammatory indices and laboratory-defined cardiac injury, liver injury, and kidney involvement, as well as hospitalization length, in children with RTIs.

We found that alterations of inflammatory indices were associated with laboratory-defined cardiac injury, liver injury, and kidney involvement. NLR, SII, and CLR, SII, NLR, and PLR, and NLR, CLR, and NPR showed a relatively high discriminative ability for laboratory-defined cardiac injury, liver injury, and kidney involvement, respectively. Higher levels of WBC, lymphocyte, CRP, NPR, and CLR, together with lower levels of platelet and Hb, were associated with longer hospitalization stay. Significant associations were observed before or/and after the infection points for the associations between neutrophils, lymphocytes, CRP, SII, NLR, PLR, NPR, and CLR, and hospitalization length.

These findings suggest that inflammatory indices may reflect systemic inflammatory and immune responses associated with laboratory-defined organ injury/involvement in children with RTIs. However, because cytokines and immune mediators were not systematically measured, the underlying biological mechanisms remain to be clarified. Several potential biological explanations may help interpret these associations, although the present study did not directly measure cytokines or immune pathways. First, increased neutrophil levels may reflect a robust early inflammatory response and can secrete cardioprotective mediators such as granulocyte colony–stimulating factor and interleukin-10 (IL-10) that clear pathogens or damaged tissue (13). This acute phase could limit the spread of injury and promote tissue repair, eventually reducing long-term myocardial dysfunction despite initial inflammation (14). Neutrophils polarize macrophages toward a reparative phenotype after myocardial infarction (15). NLR elevation is often attributable to increased neutrophils and decreased lymphocytes, and residual lymphocytes may differentiate into regulatory T cells, which secrete IL-10 and TGF-β to suppress excessive inflammation and protect myocardial function (16). Neutrophils have been shown to improve the polarization of macrophages toward a repair phenotype by releasing lipoproteins associated with neutrophil gelatinase, thereby improving heart healing after myocardial infarction (17). CRP can upregulate vascular endothelial growth factor (VEGF) expression to promote angiogenesis by activating hypoxia-inducible factor (HIF)-α via the CD64/PI3K/Akt and mitogen-activated protein kinase (MAPK)/extracellular signal-regulated kinase (ERK) pathways (18). PI3K is vital for cardiogenesis and the regulation of cardiac structure and function, and PI3K overexpression governs the development of cardiac pressure overload adaptation and compensatory hypertrophy (19). Meanwhile, the concurrent anti-inflammatory processes such as anti-inflammatory cytokine secretion during the elevation of inflammatory indices may limit excessive inflammation-induced myocardial injury. CD4+ T regulatory cells improve wound healing after myocardial infarction (20). In a word, an increase of inflammatory indices in cardiac injury is likely attributable to the adaptive inflammatory response, which promotes cardiac recovery. In this study, the participants had RTIs without severe symptoms, and the inflammatory indices were all tested within three days after hospital admission, which indicated that inflammation stimulation was likely to have been mild in the early stage. Hence, our findings were consistent with the hypotheses.

Second, the adaptive inflammatory response also limits hepatic damage by promoting debris clearance and activating endogenous repair mechanisms (21). Platelets release growth factors and anti-inflammatory mediators that stimulate liver regeneration and reduce fibrosis (22). Concurrent activation of anti-inflammatory pathways during the process of increased inflammatory response may limit excessive liver injury. PLR may also act as a protective factor in non-alcoholic fatty liver disease (23). Meanwhile, T and B lymphocytes directly or indirectly damage hepatocytes (24). Lymphocyte activation promotes chronic inflammation, fibrosis, and functional decline in liver diseases (25). Elevated lymphocytes secrete high levels of IFN-γ, TNF-α, IL-6, and IL-17, which activate hepatic stellate cells, promote fibrosis, and induce hepatocyte apoptosis (26). Lymphocyte infiltration may also disrupt liver sinusoidal blood flow and cause hypoxia and secondary hepatocyte necrosis (27). On the other hand, in sepsis or systemic inflammation, lymphocyte-derived cytokines may exacerbate liver ischemia/reperfusion injury or drug-induced liver injury (28). In a word, lymphocyte elevation may lead to liver injury directly and indirectly via various cytokines.

Third, double-negative T cells protect from early injury in moderate experimental acute kidney injury (AKI) (29). Regulatory subsets of lymphocytes suppress inflammation, clear pathogens, and promote kidney repair (30). Transferring of infiltrating lymphocytes to T-cell-deficient mice is renoprotective (31). Meanwhile, neutrophils release reactive oxygen species, proteases, and neutrophil extracellular traps, which directly damage renal tubular epithelial cells, glomeruli, and the vascular endothelium, particularly in AKI. Neutrophil extracellular traps can occlude the renal microvasculature and exacerbate ischemia and fibrosis (32). CRP can activate NF-κB, promote cytokine release, and reduce renal perfusion, which amplify inflammation, ischemic injury, and fibrosis (33). High PLR indicates platelet activation, which, in turn, recruits neutrophils to renal vessels and causes microthrombi and ischemia (34). High CLR indicates uncontrolled systemic inflammation with impaired immune regulation, wherein CRP may amplify antibody-mediated glomerular injury, while lymphocytes fail to resolve inflammation. In a word, increased WBC, neutrophils, CRP, SII, NLR, PLR, and CLR reflect a proinflammatory state that directly damages the kidneys through cytotoxicity, endothelial dysfunction, fibrosis, impaired immune regulation, and microvascular occlusion.

Finally, inflammatory indices may likely be associated with adverse outcomes, which lead to prolonged hospitalization stay.

For example, in this study, those with NLR > 12 were older males with higher disease severity and independently associated with prolonged length of stay (35). PLR and NLR were all positively associated with hospital length of stay in patients with acute exacerbation of chronic obstructive pulmonary disease (36). A preoperative CRP > 1 was an independent predictive factor for prolonged hospital stay after radical cystectomy for bladder cancer (37). A high-SII level was associated with an increased risk of longer hospitalization after orthopedic surgery (38). In a word, inflammatory indices may be associated with hospitalization length by reflecting systemic inflammatory status and clinical course. However, hospitalization length is also influenced by multiple clinical and non-clinical factors and should not be interpreted as a direct measure of disease severity.

Our findings showed associations between inflammatory indices, laboratory-defined organ injury/involvement, and hospitalization length, providing exploratory evidence for the clinical relevance of blood count–derived inflammatory indices in pediatric RTIs. Previous studies have also focused on the associations between inflammatory indices and organ injury, and some findings are consistent with our results. Han et al. reported that NLR, PLR, and SII were negatively associated with CK-MB levels, supporting the possibility that some inflammatory responses may be related to cardiac recovery (39). Joshi et al. reported that platelet activation exerted hepatoprotective effects during chronic cholestasis (40). Lisman and Porte reported that platelets stimulated liver regeneration (41) and facilitated tissue repair. Lee et al. reported that double-negative T cells had a reparative role after experimental severe ischemic acute kidney injury (29). Meanwhile, Ma et al. reported that elevated SII and hs-CRP levels were risk factors for contrast-induced AKI after PCI in patients with STEMI (42). Li et al. reported that elevated SII was independently associated with a higher risk of AKI in adults undergoing cardiac surgery (43). Wang et al. reported that the increase of hs-CRP was linked closely to the occurrence of AKI after AMI (44). Shen et al. reported that preprocedural high SII was an independent risk factor for CI-AKI after emergency PCI in patients with STEMI (45). Xie et al. reported that NLR was an independent predictor in septic patients with S-AKI (46). Wang et al. reported that the increase of hs-CRP was closely linked to the occurrence of AKI after acute myocardial infarction (44). These studies suggest that different inflammatory indices may play different roles in organ injury. The associations between inflammatory indices and RTI hospitalization length may also be related to their links with disease severity and clinical course.

Our study had several strengths. First, the investigated inflammatory indices were derived from routine blood tests and are more readily available than cytokine panels in many clinical settings, especially in primary healthcare institutions. We clarified the differentiated associations between inflammatory indices and laboratory-defined cardiac injury, liver injury, and kidney involvement, as well as the non-linear relationships between inflammatory indices and hospitalization length. These findings provide exploratory insights into the clinical relevance of easily accessible inflammatory indices in pediatric RTIs. Second, the recruited participants were generally healthy children without chronic diseases, and inflammatory indices were measured within 3 days after admission, suggesting that the observed organ injuries were likely to represent early-stage abnormalities during hospitalization. The findings, except the relationship between PLT and hospitalization length, were robust after FDR correction. This consistency suggests that most of the observed associations were robust to adjustment for multiple testing and were not materially driven by multiplicity. Nevertheless, these findings should still be interpreted as exploratory and require confirmation in future prospective studies. Third, the overall patterns of associations in the age-stratified sensitivity analyses were largely consistent with the primary analyses, suggesting that the main results were not materially driven by the inclusion of younger children or by age-related variation in laboratory reference ranges. However, several estimates within specific age strata were unstable because of limited sample size or sparse outcome events. Therefore, the age-stratified findings should be interpreted cautiously and not be considered evidence of age-specific effect modification. Finally, the inflammatory indices were derived from routine blood tests, which are easily available in healthcare institutions. Therefore, our findings may have practical value for future clinical assessment.

Several limitations should be considered. First, because of the retrospective observational design, our findings should be interpreted only as associations rather than causality. The exact interval between symptom or fever onset and laboratory data testing was not consistently available in the retrospective medical records. Inflammatory indices may vary substantially across different stages of infection, and admission timing may not accurately reflect the duration or severity of illness. Although we used laboratory data from the first available laboratory test within three days after admission to reduce variability related to hospital-based sampling, residual confounding related to disease duration before admission could not be excluded. Future prospective and longitudinal studies should standardize the timing of sampling, record the interval from symptom onset to laboratory testing, and evaluate dynamic changes in inflammatory indices during the disease course.

Second, the definitions of laboratory-defined cardiac injury, liver injury, and kidney involvement were based on routinely available laboratory indicators. These laboratory-based definitions may not fully capture the extent or severity of organ dysfunction. Of note, urine protein positivity may reflect transient renal involvement during acute infection rather than clinically confirmed acute kidney injury. Electrocardiography, echocardiography, and liver or kidney ultrasound examinations were not systematically available for all participants and therefore could not be incorporated into the outcome assessment. Future prospective studies combining laboratory, functional, and imaging examinations are needed to more accurately evaluate organ dysfunction in children with RTIs. In addition, although blood count–derived inflammatory indices may reflect systemic inflammatory and immune status, they cannot specify the underlying cytokine pathways or immune-cell functions. Cytokines such as TNF-α, IL-1β, IL-10, and interferon-related mediators were not systematically measured in all participants, and therefore, we could not directly establish a cytokine-mediated link between inflammatory indices and organ injury. Future prospective studies incorporating serial cytokine profiling, immune-cell phenotyping, and repeated organ function assessments are needed to clarify the biological mechanisms underlying these associations. Notably, hospitalization length is a complex outcome and may not be determined solely by the severity of RTIs. Unmeasured factors may influence hospitalization length, such as treatment strategies, discharge criteria, family preference, healthcare accessibility, weekend or holiday discharge delays, and hospital workflow. Therefore, the observed associations between inflammatory indices and hospitalization length should be interpreted cautiously and may be subject to residual confounding. Future prospective studies with standardized discharge criteria and detailed information on treatment and hospital management are needed to confirm these findings. In addition, age-specific reference ranges for some laboratory indicators may vary in children. Therefore, future studies using standardized pediatric criteria, repeated laboratory measurements, and more comprehensive clinical adjudication are needed to validate these findings. Third, no external validation cohort was available in this study. Therefore, the reproducibility and generalizability of these findings require confirmation in future multicenter studies. Finally, the sample size was relatively limited, which may have reduced statistical power. Therefore, studies with larger sample sizes are needed to yield more robust results. Another limitation is that hospitalization length is a complex outcome and may not be determined solely by the severity of RTIs. Although we adjusted for several clinical factors, including age, sex, preterm birth, respiratory pathogens, and pneumonia status, other unmeasured factors may also influence hospitalization length, such as treatment strategies, discharge criteria, family preference, healthcare accessibility, weekend or holiday discharge delays, and hospital workflow. Therefore, the observed associations between inflammatory indices and hospitalization length should be interpreted cautiously and may be subject to residual confounding. Future prospective studies with standardized discharge criteria and detailed information on treatment and hospital management are needed to confirm these findings.

## Conclusions

Inflammatory indices, particularly composite indices, were associated with laboratory-defined cardiac injury, liver injury, and kidney involvement, as well as hospitalization length, in children with RTIs. These findings suggest that blood count–derived inflammatory indices may serve as easily available exploratory markers for adverse clinical profiles in pediatric RTIs. Routine and dynamic monitoring of these indices may provide additional clinical information for clinical assessment, but future prospective, longitudinal, and multicenter studies incorporating functional and imaging assessments are needed to validate these associations and determine their prognostic utility.

## Data Availability

The original contributions presented in the study are included in the article/Supplementary Material, and further inquiries can be directed to the corresponding author/s.

## References

[1] Liu C Li J Deng Y Wang Q Wang X Yang S. Prevalence of six respiratory pathogens among children in Chengdu, China: a multiplex PCR-based detection study. Front Cell Infect Microbiol. (2025) 15:1690701. 10.3389/fcimb.2025.1690701 41404368 PMC12702978

[2] Khan EA Raja MH Chaudhry S Zahra T Naeem S Anwar M. Outcome of upper respiratory tract infections in healthy children: antibiotic stewardship in treatment of acute upper respiratory tract infections. Pak J Med Sci. (2020) 36(4):642–6. 10.12669/pjms.36.4.1420 32494248 PMC7260936

[3] Kruckow KL Zhao K Bowdish DME Orihuela CJ. Acute organ injury and long-term sequelae of severe pneumococcal infections. Pneumonia (Nathan). (2023) 15(1):5. 10.1186/s41479-023-00110-y 36870980 PMC9985869

[4] Goldstein SL. Acute kidney injury in children and its potential consequences in adulthood. Blood Purif. (2012) 33(1-3):131–7. 10.1159/000334143 22269297

[5] Guo Z Fan D. Editorial: The role of inflammation in organ injury. Front Immunol. (2025) 16:1717035. 10.3389/fimmu.2025.1717035 41200158 PMC12585987

[6] Cheng W Du Z Lu B. Chronic low-grade inflammation associated with higher risk and earlier onset of cardiometabolic multimorbidity in middle-aged and older adults: a population-based cohort study. Sci Rep. (2024) 14(1):22635. 10.1038/s41598-024-72988-7 39349699 PMC11442589

[7] Algul FE Kaplan Y. Increased systemic immune-inflammation Index as a novel indicator of Alzheimer’s disease severity. J Geriatr Psychiatry Neurol. (2025) 38(3):214–22. 10.1177/08919887241280880 39271460 PMC11894901

[8] Rees CA Haggie S Florin TA. Narrative review of clinical prediction models for paediatric community acquired pneumonia. Paediatr Respir Rev. (2025) 54:19–27. 10.1016/j.prrv.2025.01.002 39965990 PMC12842315

[9] Bruce CS Hoare C Mukherjee A Paul SP. Managing acute respiratory tract infections in children. Br J Nurs. (2017) 26(11):602–9. 10.12968/bjon.2017.26.11.602 28594619

[10] Skrivankova VW Richmond RC Woolf BAR Davies NM Swanson SA VanderWeele TJ. Strengthening the reporting of observational studies in epidemiology using mendelian randomization: the STROBE-MR statement. JAMA. (2021) 326(16):1614–21. 10.1001/jama.2021.18236 34698778

[11] Nascimento-Carvalho CM Souza-Marques HH. Recommendation of the Brazilian Society of Pediatrics for antibiotic therapy in children and adolescents with community-acquired pneumonia. Rev Panam Salud Publica. (2004) 15(6):380–7. 10.1590/s1020-49892004000600003 15272984

[12] Harris E. Early respiratory infections tied to premature death later in life. JAMA. (2023) 329(13):1053. 10.1001/jama.2023.3307 36920427

[13] Rosales C. Neutrophil: a cell with many roles in inflammation or several cell types? Front Physiol. (2018) 9:113. 10.3389/fphys.2018.00113 29515456 PMC5826082

[14] Ma Y. Role of neutrophils in cardiac injury and repair following myocardial infarction. Cells. (2021) 10(7):1676. 10.3390/cells10071676 34359844 PMC8305164

[15] Horckmans M Ring L Duchene J Santovito D Schloss MJ Drechsler M. Neutrophils orchestrate post-myocardial infarction healing by polarizing macrophages towards a reparative phenotype. Eur Heart J. (2017) 38(3):187–97. 10.1093/eurheartj/ehw002 28158426

[16] Wang Y Wang C Shen L Xu D. The role of regulatory T cells in heart repair after myocardial infarction. J Cardiovasc Transl Res. (2023) 16(3):590–7. 10.1007/s12265-022-10290-5 37347425

[17] Wang J. Neutrophils in tissue injury and repair. Cell Tissue Res. (2018) 371(3):531–9. 10.1007/s00441-017-2785-7 29383445 PMC5820392

[18] Chen J Gu Z Wu M Yang Y Zhang J Ou J. C-reactive protein can upregulate VEGF expression to promote ADSC-induced angiogenesis by activating HIF-1*α* via CD64/PI3k/Akt and MAPK/ERK signaling pathways. Stem Cell Res Ther. (2016) 7(1):114. 10.1186/s13287-016-0377-1 27526687 PMC4986362

[19] Ezeani M Prabhu S. PI3K signalling at the intersection of cardio-oncology networks: cardiac safety in the era of AI. Cell Mol Life Sci. (2022) 79(12):594. 10.1007/s00018-022-04627-1 36380172 PMC11803020

[20] Hofmann U Frantz S. Role of lymphocytes in myocardial injury, healing, and remodeling after myocardial infarction. Circ Res. (2015) 116(2):354–67. 10.1161/CIRCRESAHA.116.304072 25593279

[21] Xu L Wang H. A dual role of inflammation in acetaminophen-induced liver injury. Liver Res. (2023) 7(1):9–15. 10.1016/j.livres.2023.03.001 39959696 PMC11791818

[22] Shido K Chavez D Cao Z Ko J Rafii S Ding BS. Platelets prime hematopoietic and vascular niche to drive angiocrine-mediated liver regeneration. Signal Transduct Target Ther. (2017) 2:16044. 10.1038/sigtrans.2016.44 29201496 PMC5661617

[23] Yang Y He X Tan S Qu X Huang W Cai J. The association between immunoinflammatory biomarkers NLR, PLR, LMR and nonalcoholic fatty liver disease: a systematic review and meta-analysis. Clin Exp Med. (2025) 25(1):39. 10.1007/s10238-024-01539-1 39812894 PMC11735594

[24] Koda Y Kasuga R Taniki N Kanai T Nakamoto N. The impact of T cells on immune-related liver diseases: an overview. Inflamm Regen. (2025) 45(1):21. 10.1186/s41232-025-00387-0 40616144 PMC12232205

[25] Triantafyllou E Gudd CLC Possamai LA. Immune-mediated liver injury from checkpoint inhibitors: mechanisms, clinical characteristics and management. Nat Rev Gastroenterol Hepatol. (2025) 22(2):112–26. 10.1038/s41575-024-01019-7 39663461

[26] Li J Cheng L Jia H Liu C Wang S Liu Y. IFN-*γ* facilitates liver fibrogenesis by CD161+ CD4+ T cells through a regenerative IL-23/IL-17 axis in chronic hepatitis B virus infection. Clin Transl Immunology. (2021) 10(11):e1353. 10.1002/cti2.1353 34754450 PMC8563156

[27] Horie Y Wolf R Chervenak RP Jennings SR Granger DN. T-lymphocytes contribute to hepatic leukostasis and hypoxic stress induced by gut ischemia-reperfusion. Microcirculation. (1999) 6(4):267–80. 10.1111/j.1549-8719.1999.tb00110.x 10654278

[28] Guo Y Guo W Chen H Sun J Yin Y. Mechanisms of sepsis-induced acute liver injury: a comprehensive review. Front Cell Infect Microbiol. (2025) 15:1504223. 10.3389/fcimb.2025.1504223 40061452 PMC11885285

[29] Lee K Gharaie S Kurzhagen JT Newman-Rivera AM Arend LJ Noel S. Double-negative T cells have a reparative role after experimental severe ischemic acute kidney injury. Am J Physiol Renal Physiol. (2024) 326(6):F942–56. 10.1152/ajprenal.00376.2023 38634135 PMC11386976

[30] Sabapathy V Price A Cheru NT Venkatadri R Dogan M Costlow G. ST2+T-regulatory cells in renal inflammation and fibrosis after ischemic kidney injury. J Am Soc Nephrol. (2025) 36(1):73–86. 10.1681/ASN.0000000000000471 39186386 PMC11706559

[31] Weller S Varrier M Ostermann M. Lymphocyte function in human acute kidney injury. Nephron. (2017) 137(4):287–93. 10.1159/000478538 28662513

[32] Yaykasli KO Schauer C Muñoz LE Mahajan A Knopf J Schett G. Neutrophil extracellular trap-driven occlusive diseases. Cells. (2021) 10(9):2208. 10.3390/cells10092208 34571857 PMC8466545

[33] Li J Chen J Lan HY Tang Y. Role of C-reactive protein in kidney diseases. Kidney Dis. (2022) 9(2):73–81. 10.1159/000528693 PMC10090978 37065607

[34] Li L Shen Q Rao S. Association of neutrophil-to-lymphocyte ratio and platelet-to-lymphocyte ratio with diabetic kidney disease in Chinese patients with type 2 diabetes: a cross-sectional study. Ther Clin Risk Manag. (2022) 18:1157–66. 10.2147/TCRM.S393135 36597513 PMC9805708

[35] Sharma Y Thompson C Zinellu A Shahi R Horwood C Mangoni AA. The role of the neutrophil-to-lymphocyte ratio in predicting outcomes among patients with community-acquired pneumonia. Clin Med. (2025) 25(1):100278. 10.1016/j.clinme.2024.100278 PMC11731571 39672544

[36] Cai C Zeng W Wang HW Ren S. Neutrophil-to-lymphocyte ratio (NLR), platelet-to-lymphocyte ratio (PLR) and monocyte-to-lymphocyte ratio (MLR) as biomarkers in diagnosis evaluation of acute exacerbation of chronic obstructive pulmonary disease: a retrospective, observational study. Int J Chron Obstruct Pulmon Dis. (2024) 19:933–43. 10.2147/COPD.S452444 38646605 PMC11027921

[37] Hayashi T Kawashima A Ujike T Takao T Sato M Yazawa K. Preoperative CRP is a predictive factor for prolonged hospital stay after radical cystectomy for bladder cancer. BMC Urol. (2025) 25(1):162. 10.1186/s12894-025-01847-y 40634885 PMC12239416

[38] Alsabani MH Alotaibi BA Olayan LH Alghamdi AS Alshammasi MA Alqasir BA. The value of preoperative systemic immune-inflammation index as a predictor of prolonged hospital stay in orthopedic surgery: a retrospective study. Int J Gen Med. (2023) 16:4773–82. 10.2147/IJGM.S434630 37904903 PMC10613446

[39] Han Y Guo R Feng Z Wang H Li Y Zou J. Associations of systemic inflammation markers with myocardial enzymes in pediatric adenotonsillar hypertrophy: a cross-sectional study. Heliyon. (2023) 9(7):e17719. 10.1016/j.heliyon.2023.e17719 37483768 PMC10359822

[40] Joshi N Kopec AK O'Brien KM Towery KL Cline-Fedewa H Williams KJ. Coagulation-driven platelet activation reduces cholestatic liver injury and fibrosis in mice. J Thromb Haemost. (2015) 13(1):57–71. 10.1111/jth.12770 25353084 PMC4487795

[41] Lisman T Porte RJ. Mechanisms of platelet-mediated liver regeneration. Blood. (2016) 128(5):625–9. 10.1182/blood-2016-04-692665 27297793

[42] Ma K Qiu H Zhu Y Lu Y Li W. Preprocedural SII combined with high-sensitivity C-reactive protein predicts the risk of contrast-induced acute kidney injury in STEMI patients undergoing percutaneous coronary intervention. J Inflamm Res. (2022) 15:3677–87. 10.2147/JIR.S370085 35783247 PMC9241993

[43] Li Y Huang H Zhou H. Elevated postoperative systemic immune-inflammation index associates with acute kidney injury after cardiac surgery: a large-scale cohort study. Front Cardiovasc Med. (2024) 11:1430776. 10.3389/fcvm.2024.1430776 39512366 PMC11540797

[44] Wang W Xie Y Huang X Zhou Y Luo L. The value of N-terminal pro-brain natriuretic peptide and hs-CRP in predicting acute kidney injury after acute myocardial infarction. Am J Transl Res. (2022) 14(8):5501–10. PMID: 36105028. 36105028 PMC9452361

[45] Shen G He H Zhang X Wang L Wang Z Li F. Predictive value of systemic immune-inflammation index combined with N-terminal pro-brain natriuretic peptide for contrast-induced acute kidney injury in patients with STEMI after primary PCI. Int Urol Nephrol. (2024) 56(3):1147–56. 10.1007/s11255-023-03762-3 37658947

[46] Xie T Xin Q Chen R Zhang X Zhang F Ren H. Clinical value of prognostic nutritional Index and neutrophil-to-lymphocyte ratio in prediction of the development of sepsis-induced kidney injury. Dis Markers. (2022) 2022:1449758. 10.1155/2022/1449758 35711566 PMC9197608

