# Supplementary figures and images for: Differentiated associations of inflammatory indices with laboratory-defined organ injury/involvement and hospitalization length in pediatric respiratory tract infections
Source: Front Pediatr. 2026 Jul 16;14:1804507. doi: 10.3389/fped.2026.1804507 (PMC13422445; doi:10.3389/fped.2026.1804507)

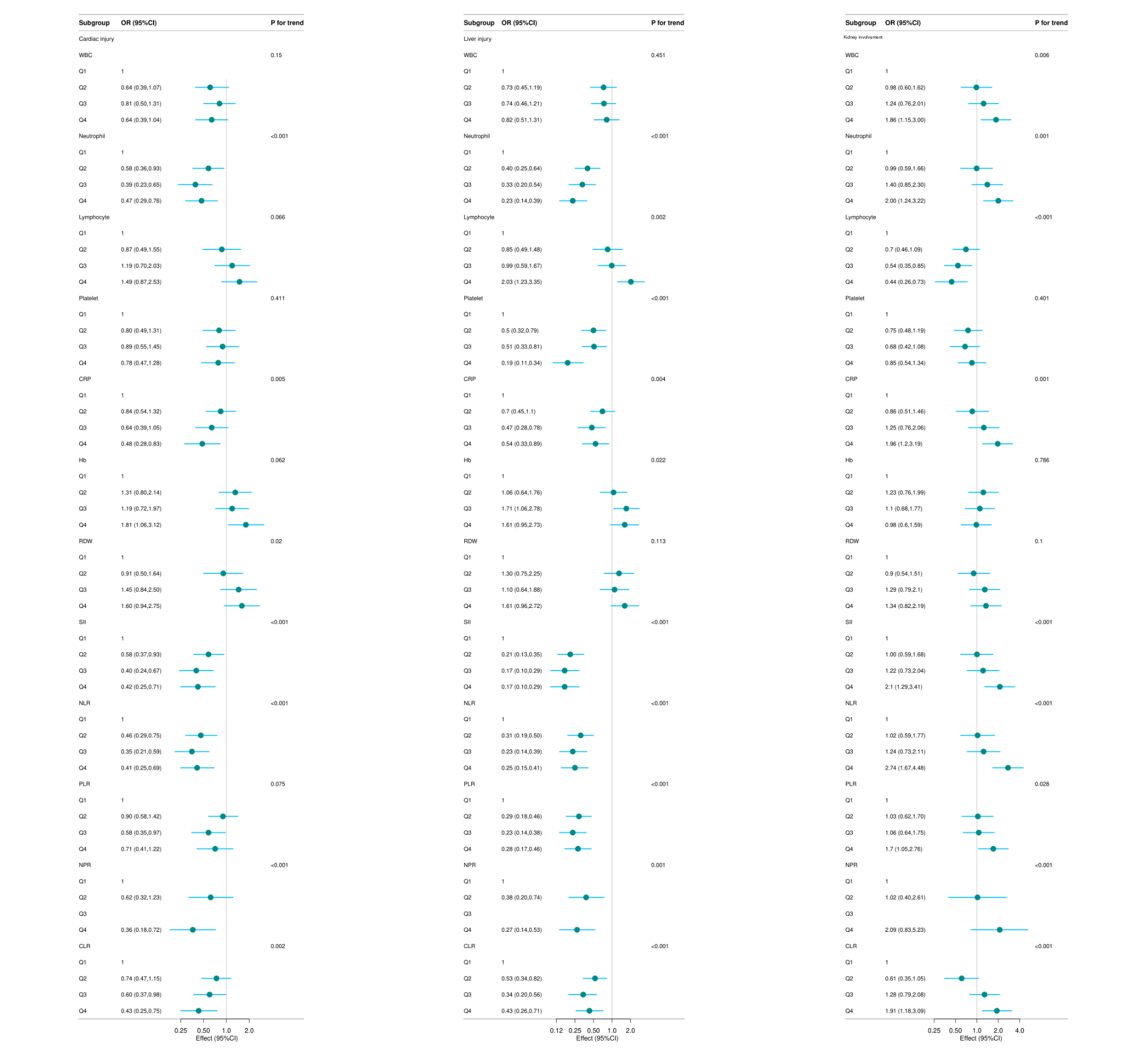

Supplement: Supplementary file 1 [file Image1.png]

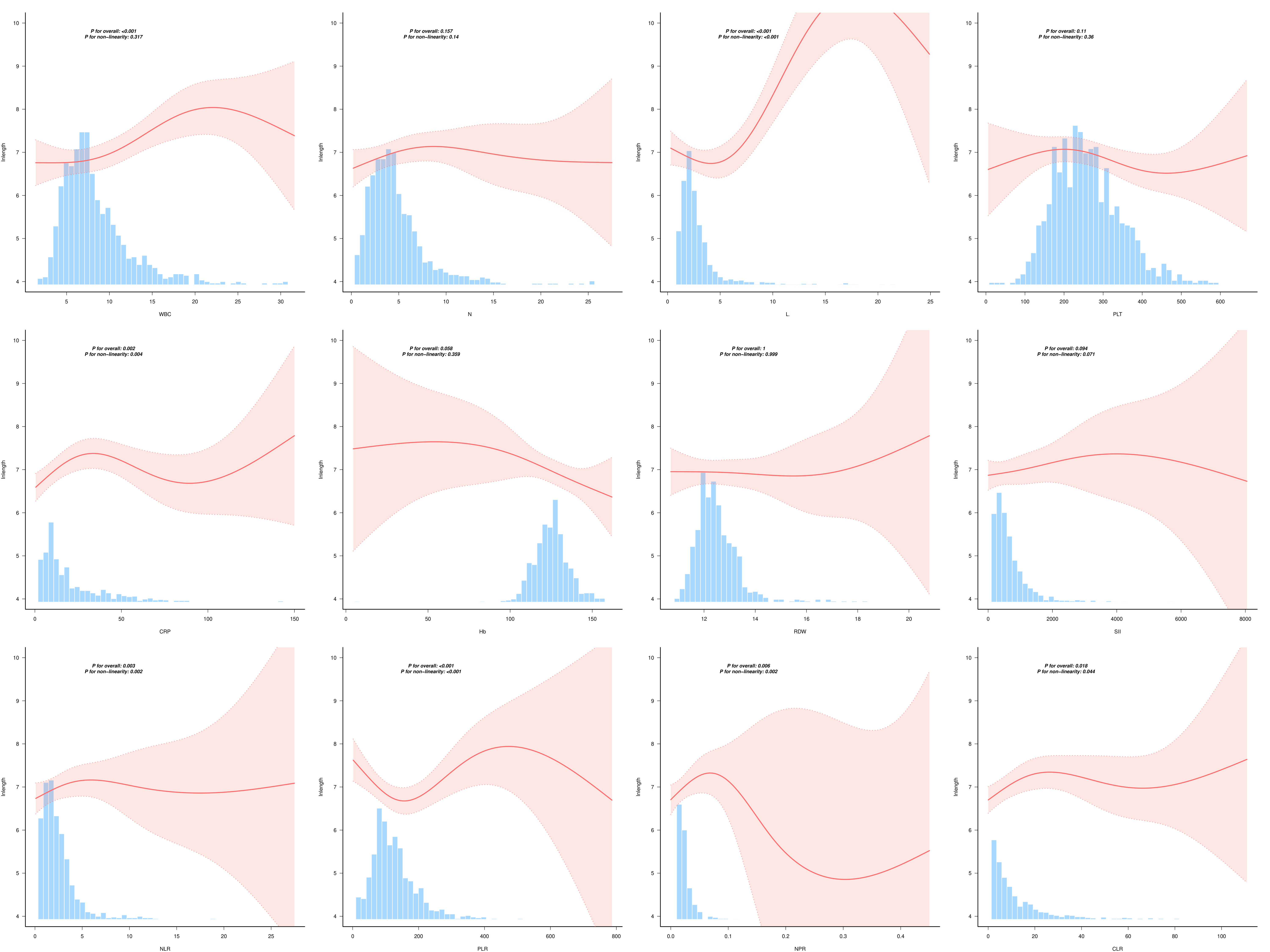

Supplement: Supplementary file 2 [file Image2.png]
