# Supplementary material for: Differentiated associations of inflammatory indices with laboratory-defined organ injury/involvement and hospitalization length in pediatric respiratory tract infections
Source: Front Pediatr. 2026 Jul 16;14:1804507. doi: 10.3389/fped.2026.1804507 (PMC13422445; doi:10.3389/fped.2026.1804507)
Supplement: Supplementary file 3 [file Supplementaryfile1.doc]

**eTable 1 Variance inflation factors for independent variables and covariates**

|  | Age | Gender | Preterm birth | FLUA | FLUB | Adenovirus | Rhinovirus | RSV | MP | Pneumonia | WBC |
| --- | --- | --- | --- | --- | --- | --- | --- | --- | --- | --- | --- |
| Variance inflation factors | 1.1 | 1 | 1.1 | 1 | 1 | 1.1 | 1 | 1.2 | 1.1 | 1.1 | 1.1 |
|  | Age | Gender | Preterm birth | FLUA | FLUB | Adenovirus | Rhinovirus | RSV | MP | Pneumonia | N |
| Variance inflation factors | 1.1 | 1 | 1.1 | 1 | 1 | 1.1 | 1 | 1.2 | 1.1 | 1.1 | 1 |
|  | Age | Gender | Preterm birth | FLUA | FLUB | Adenovirus | Rhinovirus | RSV | MP | Pneumonia | L |
| Variance inflation factors | 1.2 | 1 | 1.1 | 1 | 1 | 1.1 | 1 | 1.1 | 1.1 | 1.1 | 1.1 |
|  | Age | Gender | Preterm birth | FLUA | FLUB | Adenovirus | Rhinovirus | RSV | MP | Pneumonia | PLT |
| Variance inflation factors | 1.1 | 1 | 1.1 | 1 | 1 | 1.1 | 1 | 1.1 | 1.1 | 1.1 | 1.1 |
|  | Age | Gender | Preterm birth | FLUA | FLUB | Adenovirus | Rhinovirus | RSV | MP | Pneumonia | CRP |
| Variance inflation factors | 1.2 | 1 | 1.1 | 1 | 1 | 1.1 | 1 | 1.2 | 1.1 | 1.1 | 1.1 |
|  | Age | Gender | Preterm birth | FLUA | FLUB | Adenovirus | Rhinovirus | RSV | MP | Pneumonia | Hb |
| Variance inflation factors | 1.3 | 1 | 1.1 | 1 | 1 | 1.1 | 1 | 1.1 | 1.1 | 1.1 | 1.2 |
|  | Age | Gender | Preterm birth | FLUA | FLUB | Adenovirus | Rhinovirus | RSV | MP | Pneumonia | RDW |
| Variance inflation factors | 1.1 | 1 | 1.1 | 1 | 1 | 1.1 | 1 | 1.1 | 1.1 | 1.1 | 1 |
|  | Age | Gender | Preterm birth | FLUA | FLUB | Adenovirus | Rhinovirus | RSV | MP | Pneumonia | SII |
| Variance inflation factors | 1.2 | 1 | 1.1 | 1 | 1 | 1.1 | 1 | 1.2 | 1.1 | 1.1 | 1.1 |
|  | Age | Gender | Preterm birth | FLUA | FLUB | Adenovirus | Rhinovirus | RSV | MP | Pneumonia | NLR |
| Variance inflation factors | 1.2 | 1 | 1.1 | 1 | 1 | 1.1 | 1 | 1.2 | 1.1 | 1.1 | 1.1 |
|  | Age | Gender | Preterm birth | FLUA | FLUB | Adenovirus | Rhinovirus | RSV | MP | Pneumonia | PLR |
| Variance inflation factors | 1.2 | 1 | 1.1 | 1 | 1 | 1.1 | 1 | 1.2 | 1.1 | 1.1 | 1.2 |
|  | Age | Gender | Preterm birth | FLUA | FLUB | Adenovirus | Rhinovirus | RSV | MP | Pneumonia | NPR |
| Variance inflation factors | 1.1 | 1 | 1.1 | 1 | 1 | 1.1 | 1 | 1.2 | 1.1 | 1.1 | 1 |
|  | Age | Gender | Preterm birth | FLUA | FLUB | Adenovirus | Rhinovirus | RSV | MP | Pneumonia | CLR |
| Variance inflation factors | 1.2 | 1 | 1.1 | 1 | 1 | 1.1 | 1 | 1.2 | 1.1 | 1.1 | 1.1 |

RSV: respiratory syncytial virus, MP: mycoplasma pneumoniae, WBC: white blood cell, N: neutrophil, L: Lymphocyte, PLT: platelet, CRP: c reactive

protein, Hb: hemoglobin, RDW: red blood cell distribution width, SII: systemic immune-inflammation index, NLR: neutrophil-to-lymphocyte ratio, PLR:

platelet-to-lymphocyte ratio, NPR: neutrophil-to-platelet ratio, CLR: CRP-to-lymphocyte ratio,

**eTable 2** **Analysis of the threshold effects of inflammatory indices levels on hospitalization length**

Inflammatory indices Infection point  Coefficient (95% CI) Coefficient (95% CI) P

less than infection point more than infection point Log Likelihood Ratio Test

| WBC | 11.6 | 0.04 (-0.01, 0.10) | 0.09 (0.02, 0.15) | 0.393 |
| --- | --- | --- | --- | --- |
| Neutrophil | 4.2 | 0.16 (0.03, 0.30) | -0.01 (-0.06, 0.04) | 0.031 |
| Lymphocyte | 4 | -0.20 (-0.36, -0.05) | 0.29 (0.21, 0.36) | <0.001 |
| Platelet | 169 | 0.01 (-0.00, 0.01) | -0.00 (-0.00, -0.00) | 0.104 |
| CRP | 13.26 | 0.06 (0.03, 0.09) | -0.00 (-0.01, 0.01) | <0.001 |
| Hb | 110 | -0.00 (-0.02, 0.02) | -0.02 (-0.03, -0.00) | 0.189 |
| RDW | 11.5 | -0.96 (-3.05, 1.12) | 0.01 (-0.13, 0.16) | 0.364 |
| SII | 53.22 | -0.06 (-0.09, -0.04) | 0.00 (-0.00, 0.00) | <0.001 |
| NLR | 0.31 | -11.24 (-15.63, -6.84) | 0.07 (0.01, 0.13) | <0.001 |
| PLR | 36.39 | -0.12 (-0.15, -0.09) | 0.00 (0.00, 0.00) | <0.001 |
| NPR | 0.01 | 73.70 (14.96, 132.45) | 0.40 (-6.02, 6.83) | 0.016 |
| CLR | 9.5 | 0.06 (0.02, 0.10) | -0.00 (-0.01, 0.01) | 0.007 |

CRP: c reactive protein, Hb: hemoglobin, RDW: red blood cell distribution width, SII: aspartate aminotransferase, SII: systemic immune-

inflammation index, NLR: neutrophil-to-lymphocyte ratio, PLR: platelet-to-lymphocyte ratio, NPR: neutrophil-to-platelet ratio, CLR: CRP-to-

lymphocyte ratio, CI: confidence interval, adjust for age, gender, preterm birth, FLUA, B, Adenovirus, Rhinovirus, RSV, MP and pneumonia.

**eTable 3 Association between inflammatory indices levels and laboratory-defined cardiac and liver injury in subgroups**

Indicators Q1 Q2 Q3 Q4 P for trend

| WBC |  |  |  |  |  |
| --- | --- | --- | --- | --- | --- |
| Cardiac injury |  |  |  |  |  |
| age <3 | 1 | 1.75 (0.28, 10.88) | 2.30 (0.39, 13.39) | 1.15 (0.25, 5.35) | 0.989 |
| 3 ≤ age <6 | 1 | 0.84 (0.32, 2.20) | 0.61 (0.25, 1.52) | 0.96 (0.42, 2.19) | 0.792 |
| 6 ≤ age <12 | 1 | 0.44 (0.21, 0.90) | 0.86 (0.45, 1.66) | 0.47 (0.22, 1.03) | 0.233 |
| age ≥12 | 1 | 0.86 (0.04, 16.54) | - | 1.01 (0.05, 20.51) | - |
| 3 ≤ age | 1 | 0.50 (0.30, 0.86) | 0.70 (0.43, 1.16) | 0.72 (0.43, 1.20) | 0.362 |
| Liver injury |  |  |  |  |  |
| age <3 | 1 | 0.47 (0.08, 2.77) | 0.67 (0.13, 3.50) | 0.15 (0.03, 0.76) | 0.028 |
| 3 ≤ age <6 | 1 | 0.44 (0.15, 1.34) | 0.65 (0.25, 1.67) | 0.72 (0.30, 1.73) | 0.581 |
| 6 ≤ age <12 | 1 | 0.69 (0.34, 1.40) | 0.58 (0.27, 1.23) | 0.86 (0.42, 1.76) | 0.642 |
| age ≥12 | 1 | 2.76 (0.42, 18.27) | 3.34 (0.51, 22.04) | 4.94 (0.61, 40.02) | 0.133 |
| 3 ≤ age | 1 | 0.67 (0.40, 1.15) | 0.70 (0.41, 1.19) | 0.99 (0.60, 1.63) | 0.984 |
| Neutrophil |  |  |  |  |  |
| Cardiac injury |  |  |  |  |  |
| age <3 | 1 | 0.38 (0.09, 1.68) | 0.44 (0.08, 2.42) | 0.17 (0.03, 0.93) | 0.049 |
| 3 ≤ age <6 | 1 | 0.72 (0.29, 1.75) | 0.37 (0.14, 0.94) | 0.60 (0.26, 1.36) | 0.101 |
| 6 ≤ age <12 | 1 | 0.68 (0.34, 1.37) | 0.41 (0.19, 0.89) | 0.68 (0.33, 1.38) | 0.186 |
| age ≥12 | 1 | 0.48 (0.02, 11.48) | - | 0.63 (0.03, 14.93) | - |
| 3 ≤ age | 1 | 0.54 (0.33, 0.90) | 0.35 (0.20, 0.61) | 0.55 (0.34, 0.90) | 0.008 |
| Liver injury |  |  |  |  |  |
| age <3 | 1 | 0.29 (0.07, 1.14) | 0.18 (0.03, 1.05) | 0.11 (0.02, 0.74) | 0.008 |
| 3 ≤ age <6 | 1 | 0.16 (0.05, 0.54) | 0.39 (0.15, 1.01) | 0.27 (0.11, 0.69) | 0.007 |
| 6 ≤ age <12 | 1 | 0.56 (0.28, 1.09) | 0.37 (0.18, 0.75) | 0.28 (0.13, 0.59) | <0.001 |
| age ≥12 | 1 | 1.02 (0.23, 4.51) | 0.47 (0.08, 2.98) | 0.12 (0.01, 1.26) | 0.046 |
| 3 ≤ age | 1 | 0.39 (0.24, 0.65) | 0.36 (0.22, 0.61) | 0.25 (0.15, 0.43) | <0.001 |
| Lymphocyte |  |  |  |  |  |
| Cardiac injury |  |  |  |  |  |
| age <3 | 1 | - | - | - | - |
| 3 ≤ age <6 | 1 | 0.42 (0.13, 1.32) | 0.80 (0.30, 2.15) | 0.92 (0.36, 2.40) | 0.597 |
| 6 ≤ age <12 | 1 | 1.15 (0.58, 2.31) | 1.41 (0.71, 2.78) | 1.38 (0.63, 3.04) | 0.311 |
| age ≥12 | 1 | 1.74 (0.09, 33.92) | 1.49 (0.07, 30.99) | - | - |
| 3 ≤ age | 1 | 0.95 (0.54, 1.66) | 1.43 (0.85, 2.41) | 1.83 (1.07, 3.14) | 0.011 |
| Liver injury |  |  |  |  |  |
| age <3 | 1 | 0.44 (0.01, 17.15) | 0.32 (0.02, 5.61) | 0.21 (0.01, 3.37) | 0.203 |
| 3 ≤ age <6 | 1 | 0.23 (0.07, 0.84) | 0.55 (0.19, 1.54) | 0.58 (0.21, 1.59) | 0.835 |
| 6 ≤ age <12 | 1 | 0.93 (0.46, 1.88) | 0.63 (0.29, 1.34) | 2.29 (1.14, 4.60) | 0.066 |
| age ≥12 | 1 | 16.21 (1.10, 239.30) | 30.31 (1.75, 525.98) | - | - |
| 3 ≤ age | 1 | 0.91 (0.52, 1.60) | 1.00 (0.58, 1.74) | 2.46 (1.46, 4.15) | <0.001 |
| Platelet |  |  |  |  |  |
| Cardiac injury |  |  |  |  |  |
| age <3 | 1 | 8.51 (1.06, 68.68) | 1.45 (0.28, 7.41) | 1.64 (0.36, 7.51) | 0.994 |
| 3 ≤ age <6 | 1 | 0.48 (0.19, 1.17) | 0.70 (0.30, 1.66) | 0.55 (0.22, 1.39) | 0.320 |
| 6 ≤ age <12 | 1 | 0.87 (0.43, 1.74) | 0.88 (0.44, 1.78) | 0.75 (0.36, 1.56) | 0.471 |
| age ≥12 | 1 | - | 0.45 (0.03, 6.23) | - | - |
| 3 ≤ age | 1 | 0.68 (0.41, 1.14) | 0.82 (0.50, 1.35) | 0.71 (0.42, 1.20) | 0.281 |
| Liver injury |  |  |  |  |  |
| age <3 | 1 | 1.49 (0.28, 8.09) | 0.83 (0.18, 3.86) | 0.21 (0.05, 0.90) | 0.018 |
| 3 ≤ age <6 | 1 | 0.28 (0.11, 0.71) | 0.33 (0.13, 0.82) | 0.04 (0.01, 0.18) | <0.001 |
| 6 ≤ age <12 | 1 | 0.43 (0.22, 0.85) | 0.45 (0.23, 0.86) | 0.22 (0.10, 0.48) | <0.001 |
| age ≥12 | 1 | 0.93 (0.18, 4.67) | 0.58 (0.13, 2.52) | 0.36 (0.06, 2.27) | 0.244 |
| 3 ≤ age | 1 | 0.45 (0.27, 0.73) | 0.46 (0.28, 0.74) | 0.18 (0.10, 0.33) | <0.001 |
| CRP |  |  |  |  |  |
| Cardiac injury |  |  |  |  |  |
| age <3 | 1 | 0.45 (0.11, 1.90) | 0.11 (0.02, 0.68) | 0.36 (0.06, 2.25) | 0.063 |
| 3 ≤ age <6 | 1 | 0.76 (0.34, 1.67) | 0.93 (0.39, 2.26) | 0.39 (0.14, 1.13) | 0.148 |
| 6 ≤ age <12 | 1 | 0.83 (0.40, 1.73) | 0.74 (0.36, 1.52) | 0.59 (0.27, 1.29) | 0.173 |
| age ≥12 | 1 | - | - | - | - |
| 3 ≤ age | 1 | 0.86 (0.53, 1.40) | 0.64 (0.38, 1.06) | 0.39 (0.22, 0.69) | <0.001 |
| Liver injury |  |  |  |  |  |
| age <3 | 1 | 0.35 (0.10, 1.28) | 0.16 (0.03, 0.96) | 0.24 (0.03, 1.73) | 0.040 |
| 3 ≤ age <6 | 1 | 0.59 (0.24, 1.45) | 0.97 (0.37, 2.55) | 1.09 (0.40, 2.95) | 0.832 |
| 6 ≤ age <12 | 1 | 0.75 (0.38, 1.46) | 0.43 (0.21, 0.91) | 0.38 (0.18, 0.80) | 0.004 |
| age ≥12 | 1 | 0.77 (0.10, 6.20) | 0.10 (0.01, 1.60) | 0.72 (0.12, 4.47) | 0.790 |
| 3 ≤ age | 1 | 0.76 (0.47, 1.24) | 0.50 (0.29, 0.86) | 0.54 (0.32, 0.91) | 0.008 |
| Hb |  |  |  |  |  |
| Cardiac injury |  |  |  |  |  |
| age <3 | 1 | 2.13 (0.53, 8.59) | 1.99 (0.44, 8.96) | - | - |
| 3 ≤ age <6 | 1 | 1.40 (0.61, 3.20) | 0.92 (0.38, 2.24) | 2.06 (0.72, 5.90) | 0.428 |
| 6 ≤ age <12 | 1 | 0.90 (0.41, 1.98) | 0.87 (0.41, 1.85) | 1.14 (0.57, 2.30) | 0.671 |
| age ≥12 | 1 | - | - | - | - |
| 3 ≤ age | 1 | 1.01 (0.59, 1.71) | 0.76 (0.44, 1.30) | 0.83 (0.50, 1.38) | 0.331 |
| Liver injury |  |  |  |  |  |
| age <3 | 1 | 0.97 (0.27, 3.57) | 4.65 (0.97, 22.42) | - | - |
| 3 ≤ age <6 | 1 | 0.77 (0.30, 1.97) | 1.45 (0.60, 3.52) | 1.49 (0.49, 4.55) | 0.280 |
| 6 ≤ age <12 | 1 | 1.76 (0.77, 3.98) | 1.73 (0.78, 3.80) | 2.20 (1.04, 4.68) | 0.056 |
| age ≥12 | 1 | 0.00 (0.00, Inf) | 0.65 (0.10, 4.13) | 0.17 (0.02, 1.19) | 0.290 |
| 3 ≤ age | 1 | 1.00 (0.56, 1.76) | 1.35 (0.80, 2.29) | 1.14 (0.68, 1.92) | 0.444 |
| RDW |  |  |  |  |  |
| Cardiac injury |  |  |  |  |  |
| age <3 | 1 | 2.20 (0.35, 14.01) | 1.97 (0.38, 10.31) | 5.13 (0.93, 28.17) | 0.069 |
| 3 ≤ age <6 | 1 | 2.47 (0.59, 10.29) | 4.66 (1.18, 18.43) | 3.65 (0.93, 14.26) | 0.064 |
| 6 ≤ age <12 | 1 | 0.62 (0.29, 1.33) | 0.92 (0.45, 1.86) | 1.31 (0.64, 2.66) | 0.285 |
| age ≥12 | 1 | - | - | - | - |
| 3 ≤ age | 1 | 0.94 (0.50, 1.76) | 1.55 (0.87, 2.75) | 1.92 (1.08, 3.39) | 0.004 |
| Liver injury |  |  |  |  |  |
| age <3 | 1 | 0.21 (0.03, 1.35) | 0.61 (0.12, 3.18) | 0.30 (0.06, 1.51) | 0.356 |
| 3 ≤ age <6 | 1 | 3.22 (0.73, 14.19) | 3.74 (0.87, 16.01) | 5.14 (1.24, 21.31) | 0.027 |
| 6 ≤ age <12 | 1 | 1.60 (0.76, 3.36) | 0.88 (0.41, 1.91) | 1.58 (0.74, 3.38) | 0.577 |
| age ≥12 | 1 | 1.15 (0.15, 8.68) | 1.05 (0.14, 7.95) | 3.07 (0.44, 21.50) | 0.207 |
| 3 ≤ age | 1 | 1.66 (0.91, 3.04) | 1.27 (0.69, 2.32) | 2.23 (1.24, 4.01) | 0.020 |
| SII |  |  |  |  |  |
| Cardiac injury |  |  |  |  |  |
| age <3 | 1 | 0.47 (0.11, 2.07) | 0.10 (0.01, 0.69) | 0.14 (0.01, 1.40) | 0.014 |
| 3 ≤ age <6 | 1 | 0.95 (0.42, 2.15) | 0.56 (0.22, 1.44) | 0.52 (0.20, 1.34) | 0.109 |
| 6 ≤ age <12 | 1 | 0.42 (0.20, 0.88) | 0.41 (0.20, 0.85) | 0.44 (0.22, 0.91) | 0.048 |
| age ≥12 | 1 | 0.63 (0.03, 14.14) | 1.10 (0.05, 26.49) | - | - |
| 3 ≤ age | 1 | 0.53 (0.32, 0.88) | 0.39 (0.23, 0.66) | 0.38 (0.22, 0.65) | <0.001 |
| Liver injury |  |  |  |  |  |
| age <3 | 1 | 0.21 (0.05, 0.86) | 0.05 (0.00, 0.67) | 0.57 (0.05, 6.33) | 0.042 |
| 3 ≤ age <6 | 1 | 0.18 (0.07, 0.50) | 0.24 (0.08, 0.69) | 0.16 (0.05, 0.49) | <0.001 |
| 6 ≤ age <12 | 1 | 0.24 (0.12, 0.50) | 0.17 (0.08, 0.36) | 0.21 (0.11, 0.43) | <0.001 |
| age ≥12 | 1 | 0.16 (0.03, 0.88) | 0.17 (0.03, 1.08) | 0.03 (0.00, 0.33) | 0.003 |
| 3 ≤ age | 1 | 0.21 (0.13, 0.36) | 0.18 (0.10, 0.31) | 0.16 (0.09, 0.28) | <0.001 |
| NLR |  |  |  |  |  |
| Cardiac injury |  |  |  |  |  |
| age <3 | 1 | 0.51 (0.11, 2.28) | 0.14 (0.02, 0.94) | - | - |
| 3 ≤ age <6 | 1 | 0.78 (0.33, 1.82) | 0.56 (0.22, 1.42) | 0.61 (0.24, 1.53) | 0.194 |
| 6 ≤ age <12 | 1 | 0.47 (0.22, 0.98) | 0.41 (0.20, 0.88) | 0.46 (0.22, 0.96) | 0.074 |
| age ≥12 | 1 | - | - | 0.18 (0.01, 2.78) | - |
| 3 ≤ age | 1 | 0.43 (0.26, 0.72) | 0.33 (0.19, 0.56) | 0.37 (0.22, 0.62) | <0.001 |
| Liver injury |  |  |  |  |  |
| age <3 | 1 | 0.44 (0.10, 1.83) | 1.65 (0.25, 10.67) | 0.50 (0.02, 10.77) | 0.700 |
| 3 ≤ age <6 | 1 | 0.22 (0.08, 0.62) | 0.22 (0.07, 0.67) | 0.42 (0.16, 1.12) | 0.023 |
| 6 ≤ age <12 | 1 | 0.37 (0.18, 0.75) | 0.22 (0.11, 0.48) | 0.26 (0.12, 0.53) | <0.001 |
| age ≥12 | 1 | 0.22 (0.04, 1.28) | 0.10 (0.01, 0.69) | 0.07 (0.01, 0.47) | 0.006 |
| 3 ≤ age | 1 | 0.29 (0.17, 0.49) | 0.20 (0.11, 0.35) | 0.23 (0.13, 0.38) | <0.001 |
| PLR |  |  |  |  |  |
| Cardiac injury |  |  |  |  |  |
| age <3 | 1 | 0.78 (0.18, 3.43) | 0.22 (0.04, 1.33) | - | - |
| 3 ≤ age <6 | 1 | 0.77 (0.34, 1.72) | 0.59 (0.24, 1.44) | 0.75 (0.27, 2.06) | 0.359 |
| 6 ≤ age <12 | 1 | 1.17 (0.55, 2.48) | 0.71 (0.32, 1.58) | 0.87 (0.40, 1.89) | 0.438 |
| age ≥12 | 1 | 2.12 (0.14, 31.47) | - | - | - |
| 3 ≤ age | 1 | 0.84 (0.51, 1.37) | 0.52 (0.31, 0.89) | 0.56 (0.32, 0.95) | 0.010 |
| Liver injury |  |  |  |  |  |
| age <3 | 1 | 2.10 (0.55, 8.05) | 0.68 (0.11, 4.18) | 1.37 (0.08, 22.91) | 0.823 |
| 3 ≤ age <6 | 1 | 0.21 (0.08, 0.54) | 0.24 (0.08, 0.66) | 0.27 (0.09, 0.84) | 0.003 |
| 6 ≤ age <12 | 1 | 0.26 (0.13, 0.54) | 0.18 (0.08, 0.38) | 0.28 (0.14, 0.56) | <0.001 |
| age ≥12 | 1 | 0.00 (0.00, Inf) | 0.20 (0.04, 1.08) | 0.09 (0.02, 0.52) | 0.015 |
| 3 ≤ age | 1 | 0.21 (0.12, 0.36) | 0.19 (0.11, 0.33) | 0.22 (0.13, 0.38) | <0.001 |
| NPR |  |  |  |  |  |
| Cardiac injury |  |  |  |  |  |
| age <3 | 1 | 0.46 (0.08, 2.75) | - | 0.41 (0.06, 2.78) | - |
| 3 ≤ age <6 | 1 | 0.91 (0.30, 2.72) | - | 0.54 (0.18, 1.60) | - |
| 6 ≤ age <12 | 1 | 0.40 (0.11, 1.42) | - | 0.27 (0.08, 0.96) | - |
| age ≥12 | 1 | - | - | - | - |
| 3 ≤ age | 1 | 0.48 (0.23, 1.03) | - | 0.29 (0.14, 0.61) | - |
| Liver injury |  |  |  |  |  |
| age <3 | 1 | 0.29 (0.06, 1.36) | - | 0.35 (0.06, 1.98) | - |
| 3 ≤ age <6 | 1 | 0.21 (0.07, 0.66) | - | 0.36 (0.12, 1.04) | - |
| 6 ≤ age <12 | 1 | 0.72 (0.19, 2.76) | - | 0.41 (0.11, 1.58) | - |
| age ≥12 | 1 | - | - | - | - |
| 3 ≤ age | 1 | 0.36 (0.17, 0.80) | - | 0.26 (0.12, 0.58) | - |
| CLR |  |  |  |  |  |
| Cardiac injury |  |  |  |  |  |
| age <3 | 1 | 0.17 (0.04, 0.73) | 0.15 (0.02, 1.19) | 0.06 (0.01, 0.65) | 0.008 |
| 3 ≤ age <6 | 1 | 0.82 (0.38, 1.77) | 0.67 (0.28, 1.61) | 0.51 (0.17, 1.49) | 0.175 |
| 6 ≤ age <12 | 1 | 0.79 (0.37, 1.66) | 0.75 (0.37, 1.55) | 0.53 (0.24, 1.17) | 0.127 |
| age ≥12 | 1 | - | - | - | - |
| 3 ≤ age | 1 | 0.79 (0.48, 1.28) | 0.79 (0.48, 1.28) | 0.36 (0.20, 0.65) | <0.001 |
| Liver injury |  |  |  |  |  |
| age <3 | 1 | 0.47 (0.14, 1.54) | 0.12 (0.01, 1.47) | 0.39 (0.04, 3.82) | 0.109 |
| 3 ≤ age <6 | 1 | 0.46 (0.19, 1.13) | 0.72 (0.28, 1.83) | 1.08 (0.39, 3.06) | 0.887 |
| 6 ≤ age <12 | 1 | 0.57 (0.29, 1.11) | 0.29 (0.14, 0.62) | 0.32 (0.16, 0.67) | <0.001 |
| age ≥12 | 1 | 0.28 (0.04, 2.06) | 0.10 (0.01, 1.32) | 0.23 (0.04, 1.20) | 0.125 |
| 3 ≤ age | 1 | 0.52 (0.32, 0.85) | 0.36 (0.21, 0.62) | 0.40 (0.24, 0.67) | <0.001 |

CRP: c reactive protein, Hb: hemoglobin, RDW: red blood cell distribution width, SII: aspartate aminotransferase, SII: systemic immune-

inflammation index, NLR: neutrophil-to-lymphocyte ratio, PLR: platelet-to-lymphocyte ratio, NPR: neutrophil-to-platelet ratio, CLR: CRP-to-

lymphocyte ratio, OR: odds ratio, CI: confidence interval, Model: adjust for gender, preterm birth, FLUA, B, Adenovirus, Rhinovirus, RSV, MP and

pneumonia. A dash indicates that the estimate was not available because of sparse data, zero events, or model separation.
