# Supplementary material for: Differentiated associations of inflammatory indices with laboratory-defined organ injury/involvement and hospitalization length in pediatric respiratory tract infections
Source: Front Pediatr. 2026 Jul 16;14:1804507. doi: 10.3389/fped.2026.1804507 (PMC13422445; doi:10.3389/fped.2026.1804507)
Supplement: Supplementary file 4 [file Supplementaryfile2.docx]

**eFigure 1.** Associations between inflammatory indices quartiles and laboratory-defined cardiac injury, liver

injury and kidney involvement

**eFigure 2.** Overall and non-linear relationships between inflammatory indices levels and hospitalization length.
